# A digital twin of glimepiride for personalized and stratified diabetes treatment

**DOI:** 10.3389/fphar.2025.1686415

**Published:** 2025-10-08

**Authors:** Michelle Elias, Matthias König

**Affiliations:** Faculty of Life Science, Institute for Biology, Systems Medicine of the Liver, Humboldt-Universität zu Berlin, Berlin, Germany

**Keywords:** digital twin, diabetes, glimepiride, PBPK, physiologically based pharmacokinetic model, pharmacokinetics, personalized medicine

## Abstract

**Introduction:**

Optimizing glimepiride therapy for type 2 diabetes (T2DM) is challenged by pronounced inter-individual variability in pharmacokinetics.

**Methods:**

We developed a whole-body physiologically based pharmacokinetic (PBPK) model as a digital twin of glimepiride, enabling systematic evaluation of how patient-specific factors influence drug disposition. Using curated data from 20 clinical studies, the digital twin mechanistically simulates glimepiride’s absorption, distribution, metabolism, and excretion (ADME). It accounts for key determinants of variability including renal and hepatic function, CYP2C9 genotype, and bodyweight.

**Results:**

The model accurately reproduced observed pharmacokinetics and quantified these factors’ impact on drug exposure. Increased glimepiride exposure was predicted in individuals with hepatic dysfunction or specific CYP2C9 variants, highlighting substantial genetic and physiological effects.

**Discussion:**

This digital twin provides mechanistic insights into pharmacokinetic variability and serves as an *in silico* platform for exploring individualized dosing and patient stratification strategies, laying the foundation for clinical decision support tools to improve T2DM management.

## 1 Introduction

The global burden of type 2 diabetes mellitus (T2DM) has reached critical levels, which poses substantial health and economic challenges (American Diabetes Association Professional Practice Committee, 2024; Institute for Health Metrics and Evaluation (IHME), 2024). However, a major challenge in T2DM management is optimizing treatment, as standardized drug dosing approaches can lead to inadequate glycemic control and increase the risk of adverse events like hypoglycemia (Douros et al., 2017). To address this, personalized dosing strategies, integrating patient-specific data, are increasingly recognized as vital for improving therapeutic effect and safety (Hartmanshenn et al., 2016).

Glimepiride, a second-generation sulfonylurea, is widely used in the management of type 2 diabetes mellitus (McCall, 2001; Langtry and Glimepiride, 1998). It primarily acts by binding to the sulfonylurea receptor 1 (SUR1) subunit of ATP-sensitive potassium channels in pancreatic β -cells, which triggers channel closure, membrane depolarization, and calcium influx, ultimately stimulating insulin secretion and thereby lowering blood glucose levels (McCall, 2001; Ashcroft, 1996). Following oral administration, glimepiride achieves near-complete bioavailability (99.7%) with a half-life of 5–8 h, though its active metabolite M1 retains approximately 30% activity, prolonging glucose-lowering effects (Langtry and Glimepiride, 1998).

Despite its widespread use, glimepiride exhibits notable inter-individual variability in its pharmacokinetic (PK) and pharmacodynamic (PD) response (Yoo et al., 2011). This variability is largely driven by factors such as genetic polymorphisms in the metabolizing enzyme *CYP2C9*, as well as common comorbidities in T2DM including renal and hepatic impairment (Yoo et al., 2011; Langtry and Glimepiride, 1998; U.S. Food and Drug Administration (FDA), 1995). *CYP2C9* genetic variants, particularly *2 (Arg144Cys) and *3 (Ile359Leu) alleles, greatly reduce enzymatic activity compared to the wild-type *1, with carriers demonstrating up to 2.5-fold increased glimepiride exposure and heightened hypoglycemia risk (Suzuki et al., 2006; Yoo et al., 2011). Similarly, renal dysfunction can lead to accumulation of glimepiride metabolites, particularly the active M1 metabolite, potentially prolonging hypoglycemic effects, while hepatic impairment shows minimal impact in mild-moderate cases but may compromise *CYP2C9* activity in severe dysfunction (Rosenkranz, 1996; Rosenkranz et al., 1996). Additional factors including bodyweight (Shukla et al., 2004) and varying *CYP2C9* allele frequencies across populations further contribute to variability. Consequently, reliably predicting patient response and selecting optimal, safe glimepiride doses remains a clinical difficulty.

While empirical glimepiride pharmacokinetics models have explored aspects like genetic polymorphisms (Yoo et al., 2011), PK-PD relationships (Yun et al., 2006), diabetes-induced physiological changes (Li et al., 2015), or obesity effects (Berton et al., 2023), they address individual factors without modeling their collective effect on glimepiride pharmacokinetics. While each provides important insights, without integrated modeling they offer limited utility for dose optimization across diverse patient populations.

Physiologically based pharmacokinetic (PBPK) modeling provides a potentially powerful framework to address this challenge (Sager et al., 2015; Hartmanshenn et al., 2016; Berton et al., 2023). Unlike traditional empirical pharmacokinetic methods, PBPK simulates drug absorption, distribution, metabolism and excretion based on drug specific properties integrated with physiological systems (Hartmanshenn et al., 2016; Khalil and Läer, 2011). This allows the integration of patient-specific factors (e.g., genetics, organ function) to predict individual drug exposure (Hartmanshenn et al., 2016; Sager et al., 2015). This enables the development of a digital twin, a validated computational replica designed to mirror the drug’s behavior within specific patient populations or individuals, facilitating *in silico* pharmacokinetic prediction and personalized simulation of dosing outcomes.

This study details the development and evaluation of a whole-body PBPK model serving as a digital twin for glimepiride. Incorporating key determinants of patient variability, the model demonstrates strong predictive performance against clinical data from diverse patient groups. This digital twin serves as a quantitative tool for exploring individual therapeutic scenarios, enabling patient stratification, and laying the foundation for future clinical decision support tools.

## 2 Results

### 2.1 Glimepiride database

Clinical pharmacokinetic data from 20 studies (Table 1) were systematically curated to develop the glimepiride digital twin, encompassing diverse patient populations, dosing regimens, and physiological conditions. The workflow for the study selection is illustrated in the Supplementary Figure S1. Each study received a unique PK-DB identifier linked to its PubMed ID for traceability, and the curated dataset was made publicly available to promote transparency and reproducibility.

**TABLE 1:** Summary of studies for modeling. Overview of study identifiers, PK-DB IDs, administration routes, dosing regimens, doses (mg), co-administered drugs (*Co-admin.*), and participant characteristics, including health status, renal impairment (*Ren. imp.*), type 2 diabetes mellitus (*T2DM*), and the studied genotypes/alleles (*Allele*).

Study	PK-DB ID	Route	Dosing	Dose [mg]	Co-admin	Healthy	Ren. Imp	T2DM	Allele
Ahmed et al. (2016)	PKDB00904	oral, transdermal	single	1		✓			
Badian et al. (1994)	PKDB00907	oral, iv	single	1		✓			
Badian et al. (1996)*	PKDB00908	iv	single	1.5		✓			
Choi et al. (2014)	PKDB00903	oral	single	4	gemigliptin	✓			
US Food and Drug Administration, (1995)	PKDB00946	oral, iv	single	1, 1.5		✓			
Helmy et al. (2013)	PKDB00905	oral	single	1, 2, 3, 4, 6		✓			
Kasichayanula et al. (2011)	PKDB00924	oral	single	4	dapagliflozin	✓			
Kim et al. (2017)	PKDB00947	oral	multiple	4	rosuvastatin	✓			
Lee et al. (2012)	PKDB00948	oral	single	2		✓			*1, *3
Lehr and Damm (1990)	PKDB00949	oral	single	3		✓			
Liu et al. (2010)	PKDB00950	oral	multiple	2		✓			
Malerczyk et al. (1994)	PKDB00906	oral	single	1, 2, 4, 8		✓			
Matsuki et al. (2007)	PKDB00951	oral	single, multiple	2, 1 + 1				✓	
Niemi et al. (2002)	PKDB00952	oral	single	0.5		✓			*1, *2, *3
Ratheiser et al. (1993)	PKDB01022	iv	single	0.25, 0.5, 0.75, 1.0, 1.25, 1.5		✓			
Rosenkranz et al. (1996)	PKDB00954	oral	single, multiple	3, 1 to 8			✓	✓	
Shukla et al. (2004)	PKDB00955	oral	single	8				✓	
Suzuki et al. (2006)	PKDB00956	oral	single	1				✓	*1, *3
Wang et al. (2005)	PKDB00957	oral	single	4		✓			*1, *3
Yoo et al. (2011)	PKDB00958	oral	single	2		✓			*1, *3

^*^Metabolite M1 was administered.

### 2.2 Computational model

**FIGURE 1:**
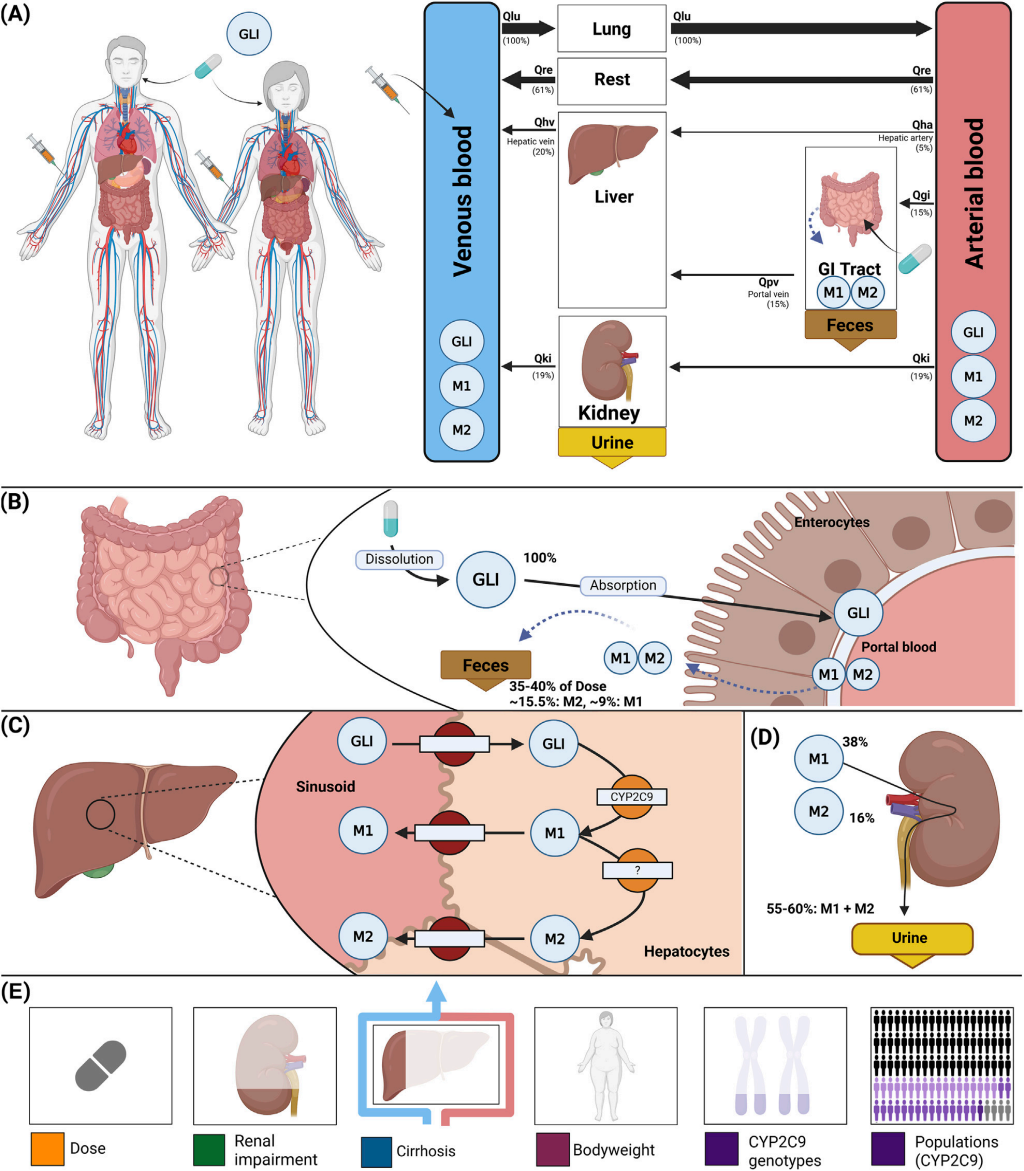
Whole-body PBPK model of glimepiride and key factors influencing its disposition. **(A)** Whole-body model illustrating glimepiride (GLI) administration (oral and intravenous), its systemic circulation via venous and arterial blood, and the key organs (liver, kidney, GI tract) involved in GLI metabolism, distribution, and excretion. **(B)** Intestinal model showing dissolution and absorption of GLI by enterocytes. No enterohepatic circulation of M1 and M2 is assumed, but reverse transport via enterocytes is included. **(C)** Hepatic model depicting CYP2C9-mediated metabolism of GLI to M1 and M2. **(D)** Renal model highlighting the elimination of M1 and M2 via urine; unchanged GLI is not excreted renally. **(E)** Key factors influencing glimepiride disposition accounted for by the model: administered dose, renal impairment, liver function (cirrhosis), bodyweight, and *CYP2C9* genotypes.

A whole-body physiologically based pharmacokinetic (PBPK) model was developed to serve as a digital twin of glimepiride, integrating key determinants of inter-individual pharmacokinetic variability (Figure 1). The model comprises key organs involved in glimepiride pharmacokinetics: gastrointestinal tract (dissolution and absorption), liver (CYP2C9-mediated metabolism to metabolites M1 and M2), and kidneys (metabolite excretion), connected via the systemic circulation. Visualizations of the submodels are provided in the supplements (Supplementary Figure S2). The digital twin incorporates patient-specific factors known to influence glimepiride pharmacokinetics: *CYP2C9* genotype variants (*1, *2, *3) through enzyme activity scaling (f_cyp2c9_), renal function impairment via glomerular filtration rate scaling (f_renal_function_), hepatic dysfunction through Child-Turcotte-Pugh score-based scaling (f_cirrhosis_), and anthropometric characteristics including bodyweight. Food effects on absorption are captured through bioavailability (f_absorption_). Model parameters were optimized against a subset of the curated clinical dataset, achieving good agreement between predictions and observed data across diverse patient populations and dosing scenarios (see Supplementary Table S1; Supplementary Figure S3 for optimized parameters). This framework enables systematic exploration of how genetic polymorphisms, organ dysfunction, and physiological characteristics influence drug exposure, providing a foundation for personalized dosing strategies. Mathematical descriptions of the model equations and ODEs for all submodels are provided in the supplements (Supplementary Section S3). Further study simulations can be found in the supplements (Supplementary Figures S9–S33).

### 2.3 Dose dependency

**FIGURE 2:**
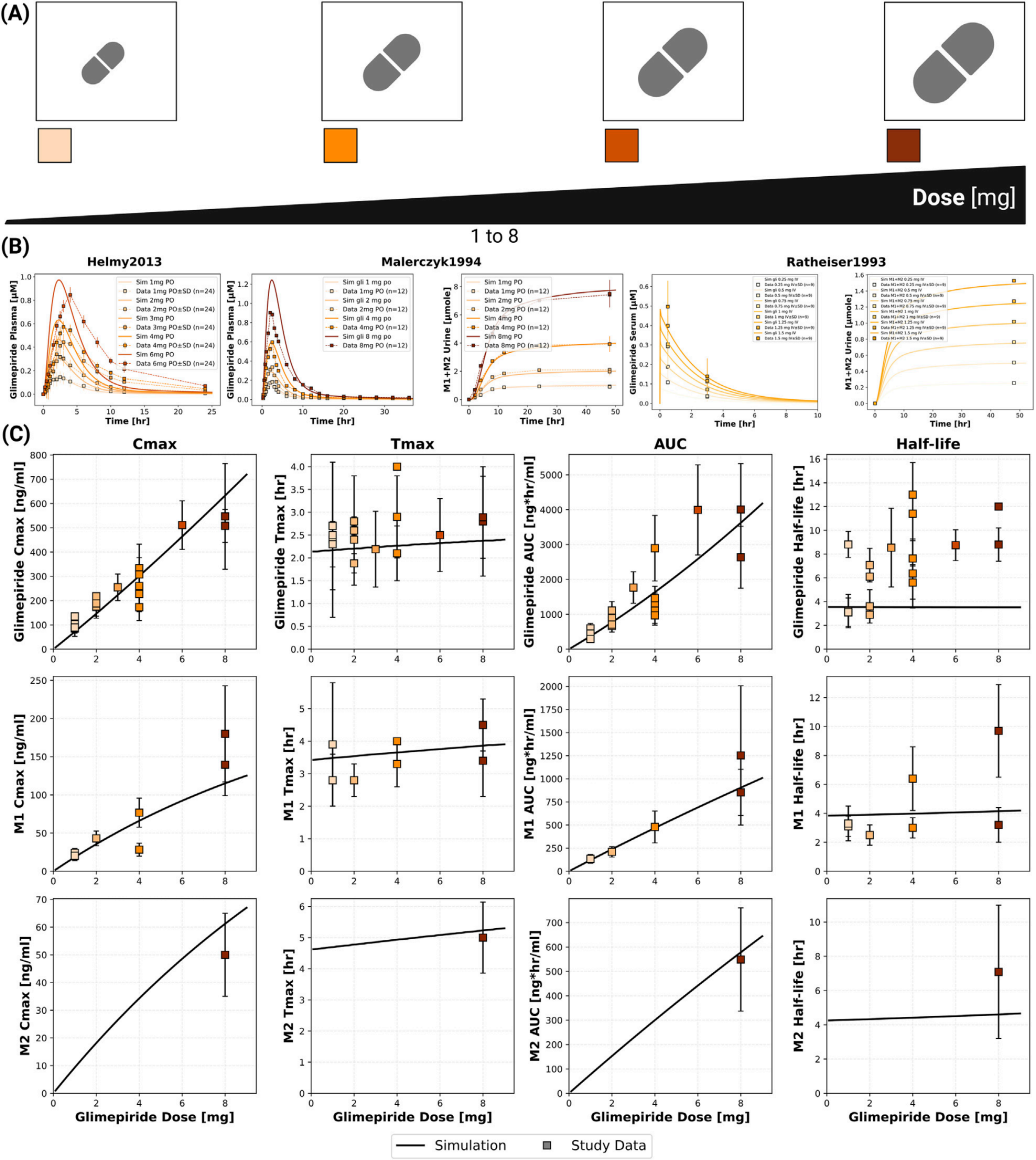
Dose dependent pharmacokinetics of glimepiride and its metabolites. **(A)** Illustration of the glimepiride oral dose range (1–8 mg) evaluated in the simulations. **(B)** Simulated (solid lines) *versus* observed (squares connected by dashed lines) plasma concentration-time profiles of glimepiride from Helmy et al. (2013), glimepiride plasma concentrations and cumulative M1+M2 urinary excretion from (Malerczyk et al., 1994 and Ratheiser et al., 1993) across various oral doses. Observed data are presented as mean or mean ± SD where available. **(C)** Dose dependency relationships for key pharmacokinetic parameters for glimepiride, M1, and M2. Simulation results (solid lines) are compared with experimental data (squares with error bars, representing mean or mean ± SD where available) aggregated from all 20 clinical studies used in the model development.

The model confirmed dose-proportional pharmacokinetics within the therapeutic dose range (1–8 mg), with C_max_ and AUC showing linear increases while T_max_ and half-life remained consistent across doses (Figure 2). Specifically, glimepiride C_max_ increased linearly from approximately 100 ng/mL at 1 mg to 700 ng/mL at 8 mg, while AUC increased proportionally from 500 to 4,000 ng*hr/mL. T_max_ remained stable at 2.0–2.5 h and half-life at approximately 4 h across all doses, confirming linear pharmacokinetics. Metabolites M1 and M2 demonstrated similar dose-proportional behavior. Simulations showed good agreement with clinical data from both Helmy 2013 (Helmy et al., 2013) and Malerczyk 1994 (Malerczyk et al., 1994) for plasma concentrations, with the model also accurately predicting cumulative urinary excretion of metabolites from Malerczyk 1994 (Malerczyk et al., 1994) (reaching about 7 μmol by 48 h for the 8 mg dose). See Supplementary Figure S4 for additional dose dependency simulations.

### 2.4 Renal impairment

**FIGURE 3:**
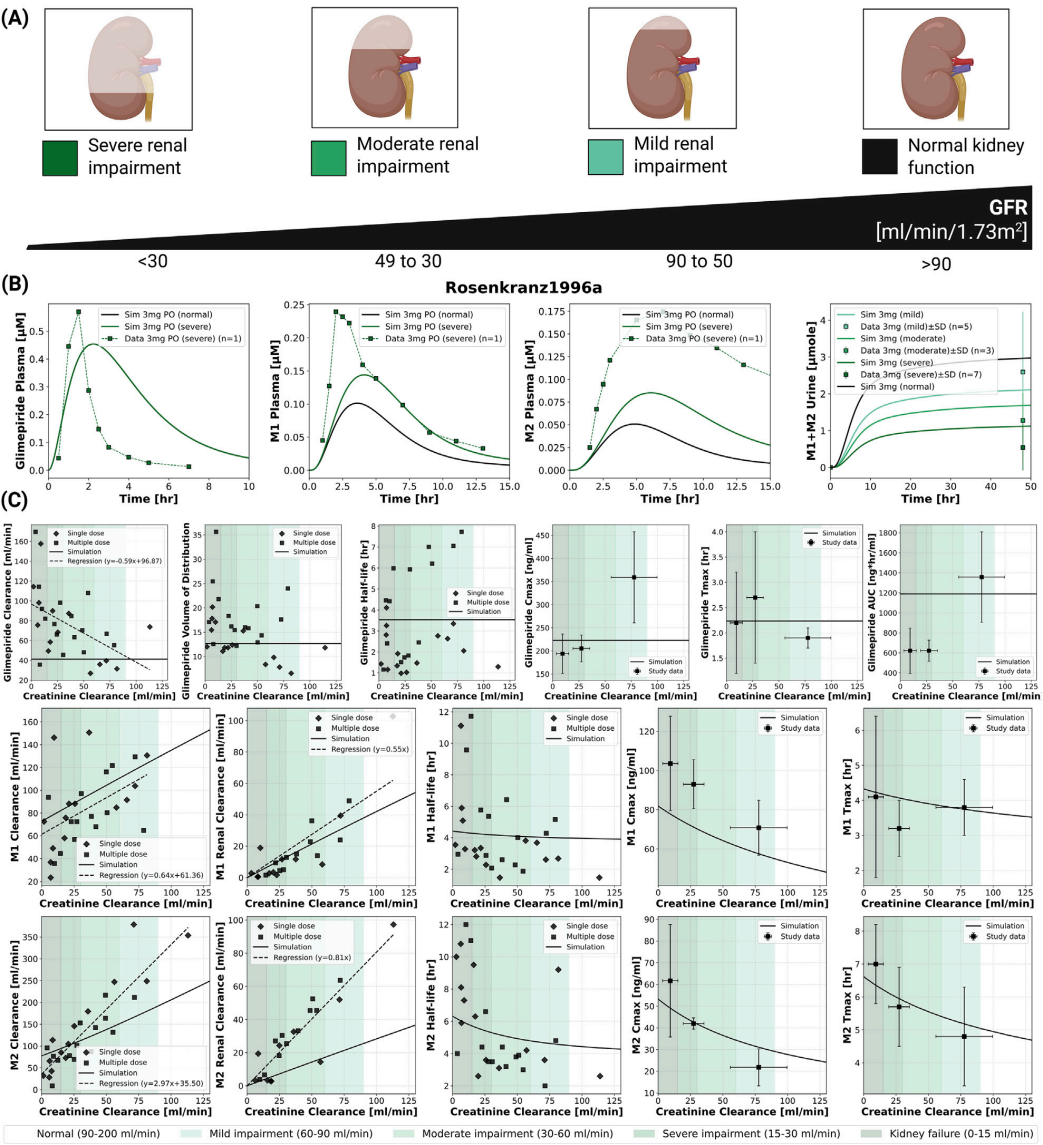
Impact of renal function on the pharmacokinetics of glimepiride and its metabolites. **(A)** Classification of renal function based on glomerular filtration rate (GFR), illustrating normal function, mild, moderate, and severe renal impairment. **(B)** Simulated (solid lines) *versus* observed (squares connected by dashed lines) plasma concentration-time profiles for glimepiride, M1, and M2, and cumulative M1+M2 urinary excretion, following a 3 mg oral dose in subjects with varying degrees of renal function. Observed data from (Rosenkranz et al., 1996). **(C)** Relationship between creatinine clearance and key pharmacokinetic parameters for glimepiride, M1, and M2, following a 3 mg oral dose. Simulation results (solid lines) are compared with observed clinical data (symbols; dashed lines: regression fits where applicable) from (Rosenkranz et al., 1996).

The model incorporated four categories of renal function based on glomerular filtration rate [mL/min/1.73 m^2^]: normal ( > 90), mild impairment (50–90), moderate impairment (35–49), and severe impairment ( < 35) based on current KDIGO guidelines (Stevens et al., 2024). Renal dysfunction primarily affected metabolite clearance with unchanged parent drug exposure (Figure 3). Simulations accurately reproduced clinical observations from (Rosenkranz et al., 1996). While the model predicted stable glimepiride clearance, the clinical data showed an apparent increase in clearance with declining renal function. In contrast, metabolites M1 and M2 showed progressive accumulation with worsening renal function, with M1 clearance declining from approx. 140 mL/min in normal function to 50 mL/min in severe impairment, and M2 clearance dropping from 250 mL/min to 70 mL/min. The cumulative urinary excretion of metabolites decreased from approx. 3 μ mol at 48 h in normal function to 1 μ mol in severe impairment following a 3 mg dose. This effect confirms the unchanged dosing requirements in renal impairment, though M1 accumulation may be relevant for any residual pharmacological activity. See Supplementary Figure S5 for additional renal impairment simulations.

### 2.5 Hepatic impairment

**FIGURE 4:**
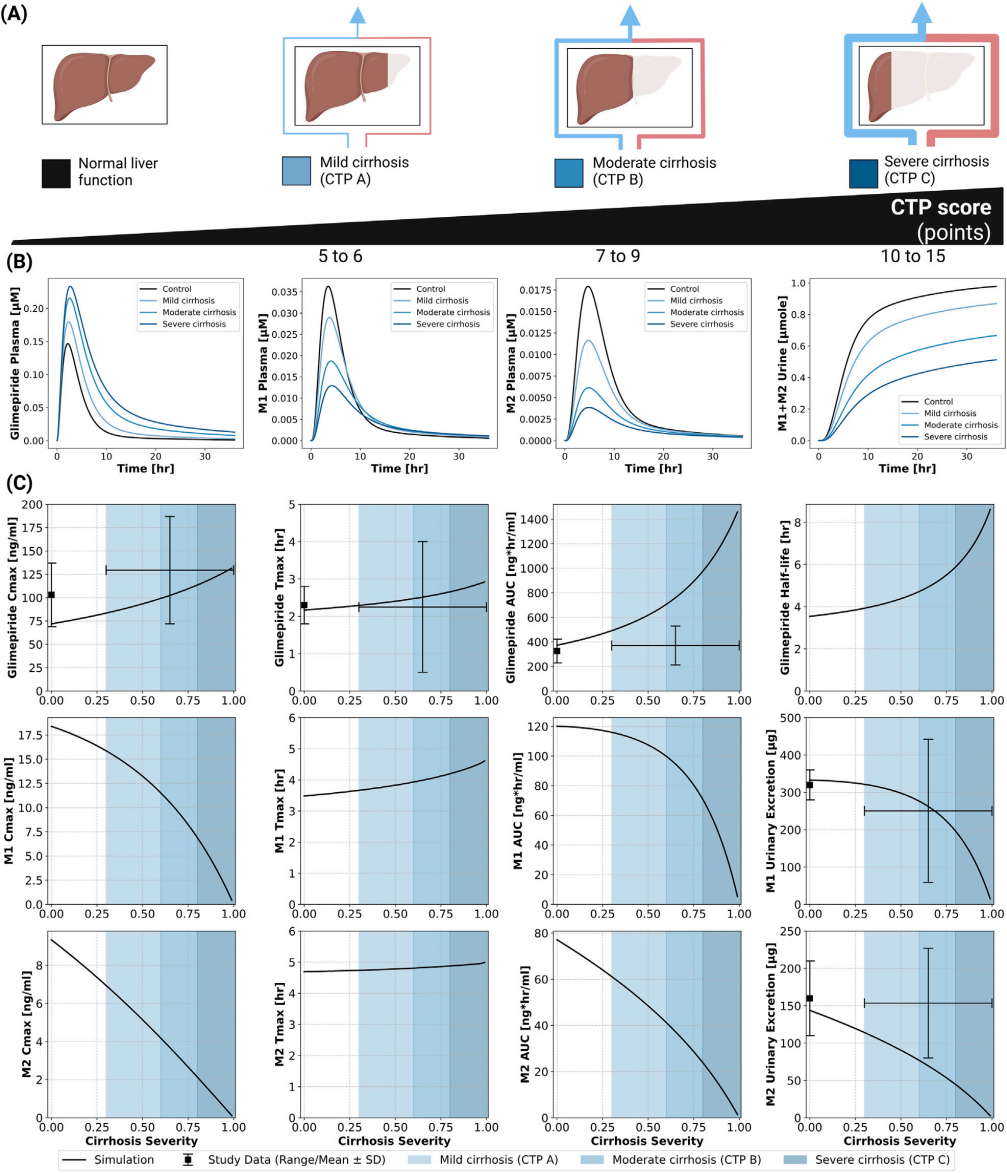
Impact of hepatic function (cirrhosis) on the pharmacokinetics of glimepiride and its metabolites. **(A)** Classification of liver function based on the Child-Turcotte-Pugh (CTP) score, illustrating normal function, mild cirrhosis (CTP A), moderate cirrhosis (CTP B), and severe cirrhosis (CTP C). **(B)** Simulated plasma concentration-time profiles for glimepiride, M1, and M2, and cumulative M1+M2 urinary excretion, following a 1 mg oral dose in subjects with varying degrees of cirrhosis severity (control, mild, moderate, severe). **(C)** Relationship between cirrhosis severity and key pharmacokinetic parameters for glimepiride, M1, and M2, following a 1 mg oral dose. Simulation results (solid lines) are compared with observed clinical data (symbols with error bars where available, representing range/mean ± SD) from (Rosenkranz, 1996).

The model incorporated Child-Turcotte-Pugh (CTP) classifications: CTP A (mild cirrhosis, 5–6 points), CTP B (moderate cirrhosis, 7–9 points), and CTP C (severe cirrhosis, 10–15 points) (Child and Turcotte, 1964; Infante-Rivard et al., 1987). Hepatic dysfunction demonstrated a strong impact on parent drug exposure (Figure 4). Model predictions matched limited clinical data, showing progressive increases in glimepiride concentrations with worsening liver function. C_max_ nearly doubled from 75 ng/mL in normal function to 125 ng/mL in severe cirrhosis, while AUC increased even more substantially by approximately 3.5-fold. Conversely, metabolite concentrations decreased greatly, reflecting reduced CYP2C9-mediated metabolism due to liver impairment. Comparison with limited clinical data from Rosenkranz 1996 (Rosenkranz, 1996) showed reasonable agreement. These findings strongly support dose reduction recommendations in hepatic impairment. See Supplementary Figure S6 for additional hepatic impairment simulations.

### 2.6 Bodyweight dependency

**FIGURE 5:**
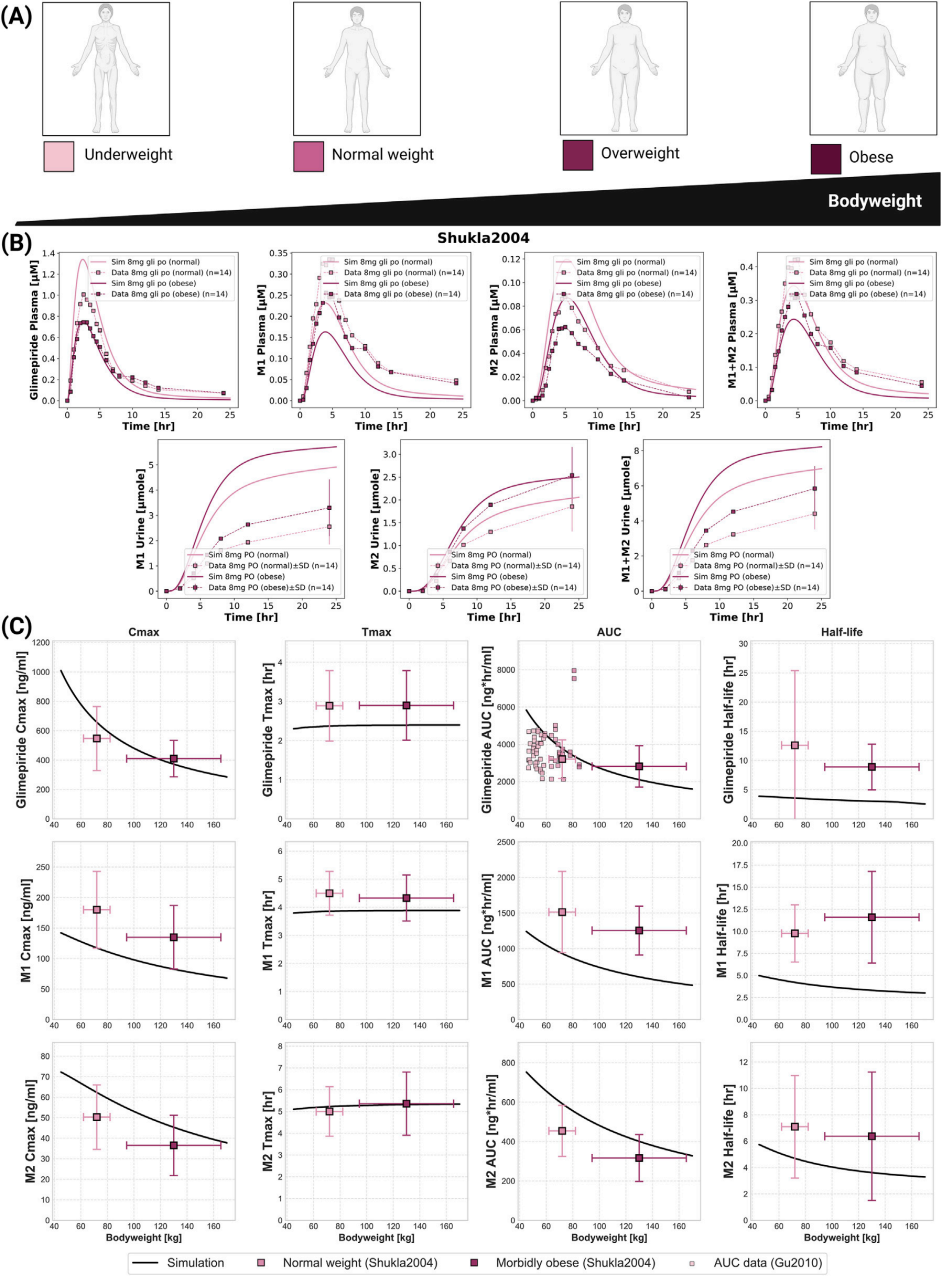
Impact of bodyweight on the pharmacokinetics of glimepiride and its metabolites. **(A)** Illustration of bodyweight categories: underweight, normal weight, overweight, and obese. **(B)** Simulated (solid lines) *versus* observed (squares connected by dashed lines) plasma concentration-time profiles and cumulative urinary excretion for glimepiride, M1, and M2, following an 8 mg oral dose in normal weight and morbidly obese individuals. Observed data from (Shukla et al., 2004). **(C)** Relationship between bodyweight and key pharmacokinetic parameters for glimepiride, M1, and M2, following a 8 mg oral dose. Simulation results (solid lines) are compared with observed clinical data (mean ± SD) from (Shukla et al., 2004) (8 mg PO, normal weight and morbidly obese groups) and dose-normalized AUC data for glimepiride from (Gu et al., 2010) (original 2 mg PO scaled to 8 mg).

An inverse relationship between bodyweight and systemic exposure was confirmed through simulations across a wide weight range (40–170 kg) and compared against clinical studies (Figure 5). Glimepiride C_max_ decreased from 1,000 ng/mL at 40 kg to 300 ng/mL at 170 kg, while AUC declined from 6,000 to approximately 2,000 ng*hr/mL. Despite these exposure changes, T_max_ and half-life remained stable across the weight range. Model predictions accurately captured observed differences between normal-weight and morbidly obese patients in (Shukla et al., 2004), with peak concentrations of 1.4 μ g/mL in normal-weight *versus* 0.8 μ g/mL in obese individuals following an 8 mg dose. Metabolites showed similar behavior. Additional comparison using AUC data from (Gu et al., 2010) further confirmed the model’s accuracy. These findings show exposure differences that may explain variable glycemic responses in obese patients, suggesting bodyweight may be an underappreciated factor in dosing practices. See Supplementary Figure S7 for additional bodyweight simulations.

### 2.7 CYP2C9 polymorphisms

**FIGURE 6:**
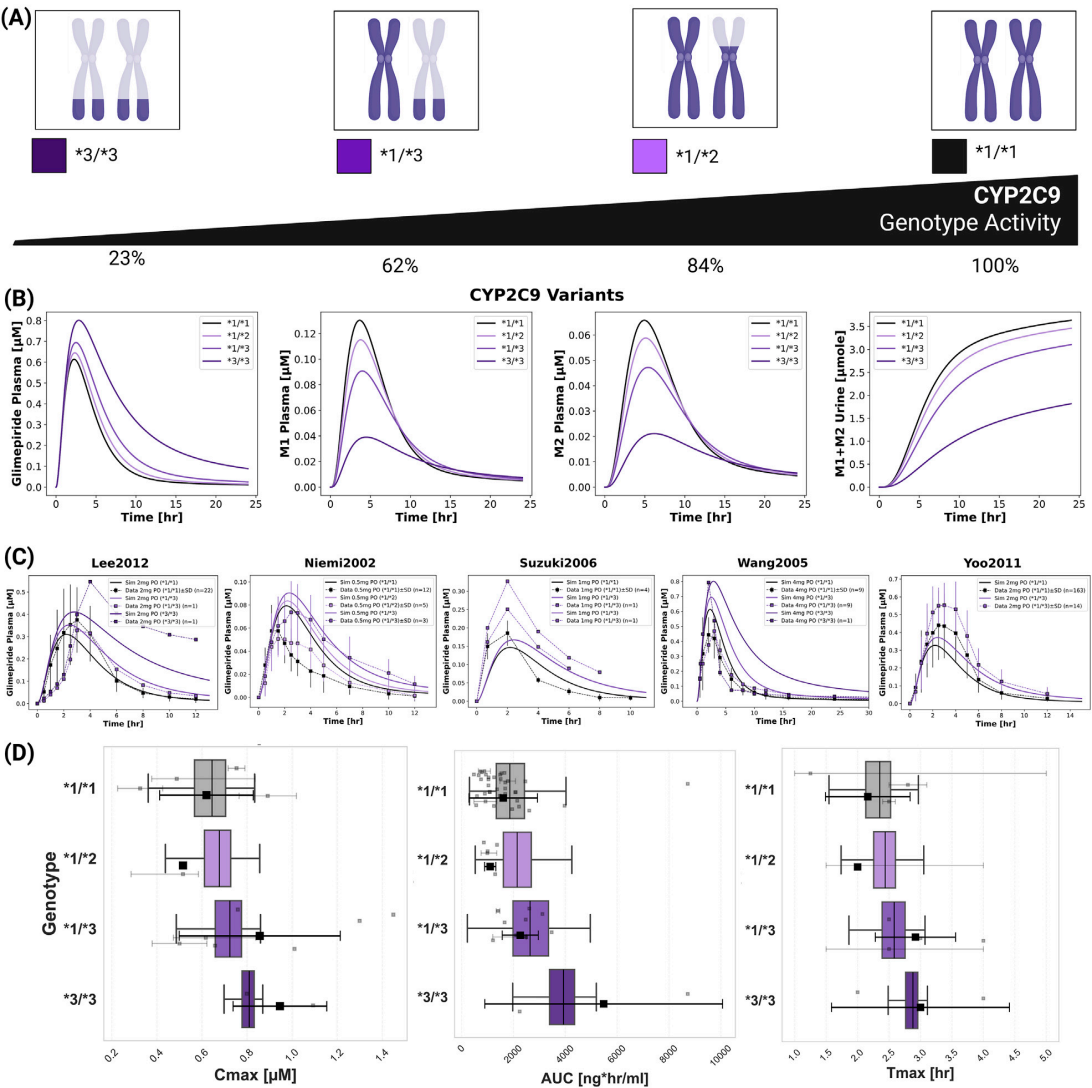
Impact of *CYP2C9* genetic variants on glimepiride pharmacokinetics. **(A)** Illustration of key *CYP2C9* genotypes (*1/*1, *1/*2, *1/*3, *3/*3) and their corresponding enzymatic activities. **(B)** Simulated pharmacokinetic profiles of glimepiride, M1, M2, and cumulative M1+M2 urinary excretion, following a 4 mg oral dose, based on fixed enzyme activity values for different *CYP2C9* genotypes. **(C)** Comparison of simulated (solid lines, using fixed *CYP2C9* activity values) *versus* observed (symbols connected by dashed lines) glimepiride plasma concentrations in individuals with different *CYP2C9* genotypes across five clinical studies (Lee et al., 2012; Niemi et al., 2002; Suzuki et al., 2006; Wang et al., 2005 and Yoo et al., 2011). **(D)** Boxplots comparing simulated glimepiride pharmacokinetic parameters derived from the probabilistic sampling approach (colored boxplots) with observed clinical data (grey squares: individual data points; black squares: weighted arithmetic mean) across different *CYP2C9* genotypes. Simulations correspond to a 4 mg oral dose. Observed data was aggregated from the clinical studies cited and dose-scaled to 4 mg where necessary.

*CYP2C9* genetic polymorphisms showed the most pronounced impact on individual pharmacokinetics (Figure 6). The model incorporated allele-specific enzyme activities (*1 = 100%, *2 = 68%, *3 = 23%), resulting in diplotype activities of 100% (*1/*1), 84% (*1/*2), 62% (*1/*3), and 23% (*3/*3). Supplementary Table S2 contains all CYP2C9 allele activities derived from literature. Simulations accurately captured substantially increased glimepiride exposure in carriers of reduced-function alleles with *3/*3 homozygotes showing up to 2.5-fold higher AUC compared to wild-type carriers. Metabolites displayed inverse patterns, with reduced formation and excretion in poor metabolizers. Model predictions demonstrated good agreement across five clinical studies (Lee et al., 2012; Yoo et al., 2011; Niemi et al., 2002; Suzuki et al., 2006; Wang et al., 2005) with doses ranging from 0.5 to 4 mg. See Supplementary Figure S8 for additional *CYP2C9* polymorphism simulations. A probabilistic modeling approach incorporating lognormal distributions of enzyme activity within genotypes captured inter-individual variability more realistically than fixed scaling factors. Supplementary Tables S3,S4 summarize the intrinsic clearance data and the fitted lognormal distribution used in the probabilistic modeling approach. This approach successfully reproduced the observed variability in pharmacokinetic parameters across genotypes. Supplementary Table S5–8 summarize the probabilistically sampled CYP2C9 allele activities, and genotype-specific glimepiride, M1 and M2 pharmacokinetics.

### 2.8 Populations

**FIGURE 7:**
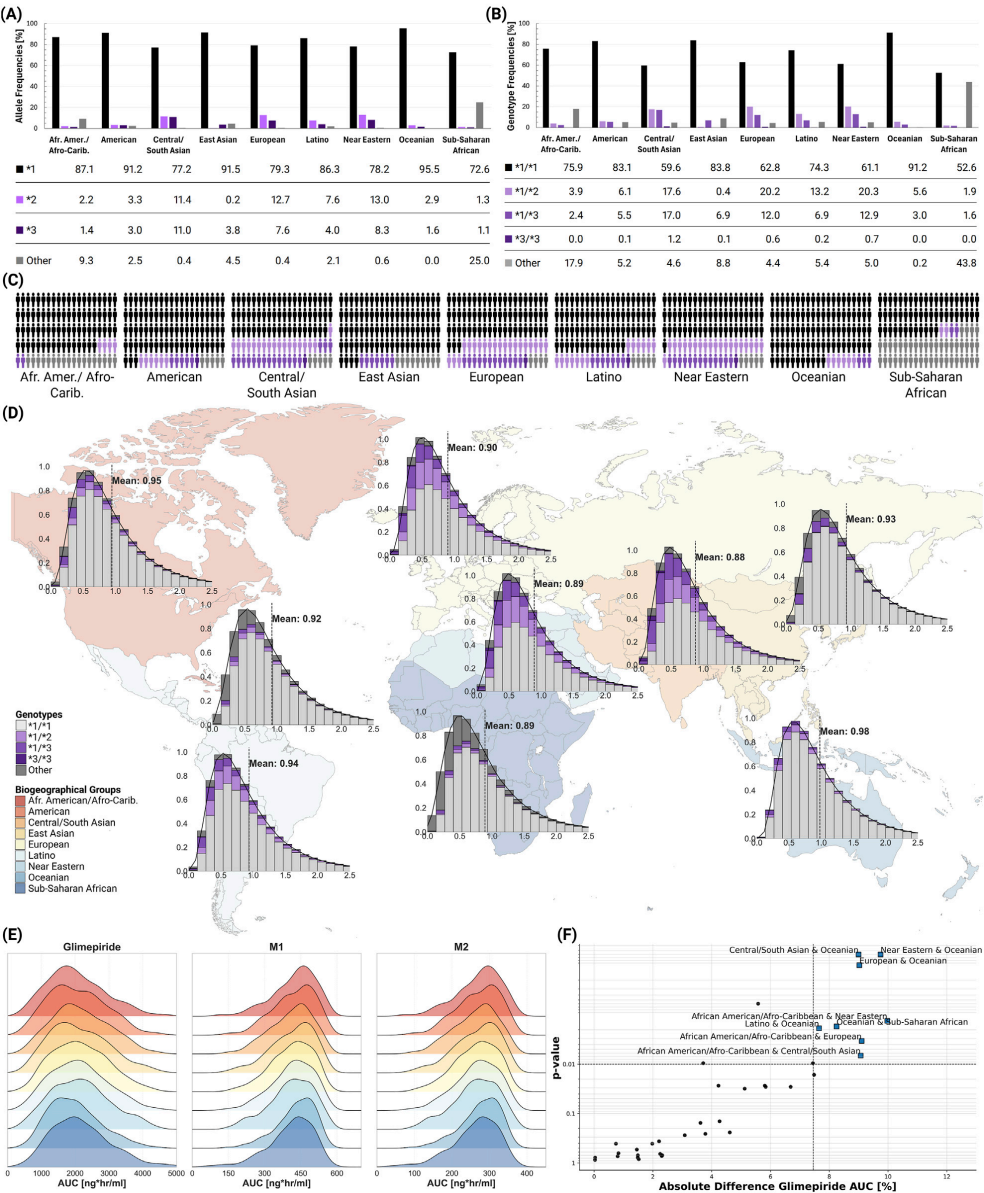
Global *CYP2C9* genetic variability and population-level impact on glimepiride pharmacokinetics. **(A)** *CYP2C9* allele frequencies across biogeographical groups (ClinPGx, 2025), showing the distribution of key alleles. **(B)** *CYP2C9* genotype frequencies across biogeographical groups (ClinPGx, 2025), showing the distribution of key genotypes. **(C)** Individual genetic variability representation within each biogeographical group. **(D)** World map displaying population-specific *CYP2C9* activity distributions derived from allele frequencies, with kernel density estimation (KDE) curves and mean enzymatic activity values shown for each biogeographical group. **(E)** Ridgeline plots comparing glimepiride, M1, and M2 AUC distributions across biogeographical populations. **(F)** Statistical comparison of population pairs showing the relationship between significance and magnitude of pharmacokinetic differences, with some comparisons showing statistically significant but clinically modest differences in glimepiride AUC.

Population-level simulations incorporating known genotype frequencies across biogeographical groups revealed modest differences in average *CYP2C9* activity and pharmacokinetic parameters between populations, despite varying genotype frequencies (Figure 7). The *2 allele showed highest frequencies in European (12.7%) and Central/South Asian (11.4%) populations, while the *3 allele was most prevalent in Central/South Asians (11.0%). Mean *CYP2C9* activity ranged from 0.88 in Central/South Asian to 0.98 in Oceanian populations. Despite these differences in genetic makeup, ridgeline plots of AUC distributions showed substantial overlap across all populations. While Kolmogorov-Smirnov testing identified statistically significant differences between certain population pairs (e.g., Central/South Asian and Oceanian, Near Eastern and Oceanian, European and Oceanian; all p < 0.01), the clinical magnitude remained small with mean differences less than 10%. Supplementary Table S9–13 provide results of the population-level simulations, including AUC, C_max_, and T_max_ values for glimepiride and its metabolites across biogeographical groups, significant pairwise differences, and sampled genotype frequencies.

### 2.9 Web application

**FIGURE 8:**
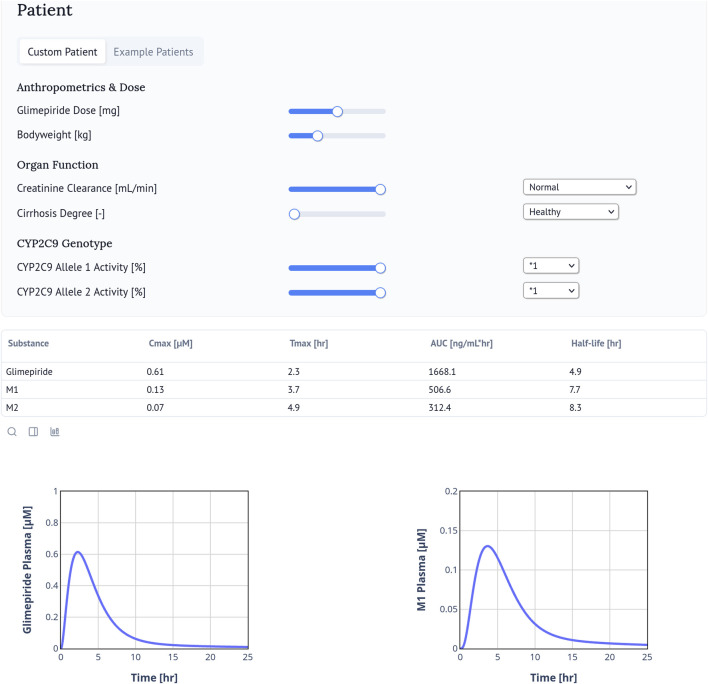
Glimepiride Web Application. The glimepiride digital twin web application simulates the pharmacokinetics of glimepiride using the PBPK model. Users can input patient-specific parameters such as bodyweight, renal or hepatic impairment, and *CYP2C9* genotype. The web application visualizes individual drug time-course profiles and calculates and displays key pharmacokinetic parameters including $C_{\text{max}}$ , $T_{\text{max}}$ , AUC, and half-life. The interface allows interactive exploration of model predictions under various clinical scenarios. The web application is available at https://glimepiride.de.

The web application of the digital twin enables real-time simulation and visualization of plasma concentration-time profiles for glimepiride and its metabolites (M1, M2) based on individual patient characteristics (Figure 8). Users can simulate personalized pharmacokinetic profiles by adjusting clinically relevant parameters and accessing calculated values for C_max_, T_max_, AUC, and half-life. Freely accessible at https://glimepiride.de, the tool supports interactive exploration of model-informed variability in drug exposure.

## 3 Discussion

In this study, we developed a whole-body PBPK model as a digital twin for glimepiride, integrating key patient-specific factors like organ function, bodyweight, and *CYP2C9* genetics. The model accurately reproduced glimepiride pharmacokinetics across diverse clinical scenarios, providing a quantitative framework to explore the drivers of variability and support personalized dosing strategies for type 2 diabetes.

The digital twin quantifies the influence of various patient factors, enabling patient stratification. It provides a quantitative platform that guides the personalization of glimepiride therapy and supports clinical decisions on initial dosing to ensure patient safety. A key strength of this PBPK approach is its ability to integrate multiple patient factors simultaneously. Unlike traditional studies that often isolate single variables, our integrated model more accurately reflects the complex clinical reality where patients present with multiple conditions affecting drug disposition. This framework is especially valuable for evaluating pharmacokinetic risks in underrepresented populations or complex scenarios where clinical evidence is lacking, providing a robust platform to support dosing decisions.

While our model confirms that glimepiride exposure is unaffected by renal impairment, it highlights the clinical significance of metabolite accumulation. The progressive buildup of the active M1 metabolite, which retains approximately 30% of the parent compound’s hypoglycemic activity, suggests a risk of prolonged adverse effects in patients with severe renal dysfunction. Rosenkranz et al. (1996) reported an apparent increase in glimepiride clearance with declining renal function, which may be explained by reduced albumin binding in chronic kidney disease. Lower albumin levels, structural modifications, and competing uremic toxins could increase the unbound drug fraction available for hepatic metabolism (Reidenberg and Drayer, 1984; Gibaldi, 1977; Gibson, 1986). Our model does not yet account for these protein binding changes, representing an area for future refinement. Therefore, although glimepiride dose adjustments may not be required, enhanced glycemic monitoring is warranted in this population. The model’s characterization of metabolite disposition was constrained by limited public data on elimination pathways and the specific enzymes responsible for M1-to-M2 conversion, with the M2 hepatic export parameter reaching its optimization boundary, yet provided physiologically reasonable predictions across diverse renal function states.

In contrast to renal function, hepatic impairment substantially increased glimepiride exposure by hindering its CYP2C9-mediated metabolism, with a progressive increase in plasma concentrations corresponding to worsening cirrhosis severity. This was accompanied by reduced metabolite formation, an inverse relationship that directly reflects impaired hepatic drug metabolism. Standard doses in patients with moderate to severe cirrhosis could lead to a significant risk of hypoglycemia. Current clinical guidelines are qualitative, only advising caution. Our digital twin provides a quantitative tool that addresses this issue by enabling *in silico* evaluation of dose adjustments needed to maintain safety in this vulnerable population.

The model demonstrated an inverse relationship between bodyweight and glimepiride exposure, with both C_max_ and AUC decreasing with increasing bodyweight while elimination kinetics remained constant. This suggests bodyweight predominantly influences volume of distribution rather than clearance, aligning with clinical observations in obese patients. This understanding supports the current clinical practice, where this level of variability is effectively managed by titrating the dose according to a patient’s glycemic response, rather than adhering to weight-based protocols.

*CYP2C9* genetic polymorphism substantially influences glimepiride exposure, with carriers of the *3/*3 genotype exhibiting approximately two-fold higher AUC compared to wild-type individuals. Despite lacking glimepiride-specific enzyme kinetic data, the model successfully leveraged CYP2C9 clearance data from related substrates to predict genotype effects, demonstrating a key strength of mechanistic PBPK approaches. Those with reduced-function alleles are at a higher risk of experiencing adverse events from a standard dose. However, our analysis shows substantial pharmacokinetic variability even within the same genotype group, with considerable overlap between different genotypes. This indicates that genotype alone is not a good predictor of patient response. Furthermore, although genotype effects were evident at the individual level, the model predicted only modest differences in pharmacokinetics across biogeographical populations. Therefore, ethnicity alone provides limited value for guiding dosing decisions. A targeted genotyping strategy focusing on patients with poor glycemic control or adverse effects may be more cost-effective than universal screening.

This digital twin of glimepiride successfully quantifies the impact of genetics, organ function, and physiology on pharmacokinetic variability and lays the basis for future clinical decision support tools that can guide personalized initial dosing, especially for patients with high-risk profiles. To facilitate clinical translation and educational use, we deployed the model as an interactive web application that enables real-time simulation of patient-specific pharmacokinetic profiles. This tool allows clinicians and researchers to explore how different patient characteristics influence drug exposure, providing immediate visual feedback for various clinical scenarios. While the specific parameters and pathways are unique to glimepiride, the modeling framework and approach demonstrated here could inform the development of similar digital twins for other medications where inter-patient variability poses clinical challenges, particularly other sulfonylureas that share CYP2C9-mediated metabolism and similar organ function dependencies. Future work should focus on refining the model using larger population studies and expanding its application to include pharmacodynamics between drug exposure and glycemic response. However, this requires dedicated clinical studies that simultaneously capture detailed PK profiles and glycemic outcomes–data that are currently limited in the literature. Another potential refinement would be to incorporate sex-specific physiological differences. Currently, our mean model does not differentiate between sex because most glimepiride clinical pharmacokinetics studies were conducted in male or mixed cohorts without reporting sex-stratified results. Although the FDA label (U.S. Food and Drug Administration (FDA), 1995) reports no sex-related differences in glimepiride pharmacokinetics after adjusting for bodyweight, more balanced datasets would allow future modeling efforts to address potential sex-related effects. As precision medicine advances, such digital twin approaches have clear potential to become valuable tools for optimizing drug therapy in complex diseases like type 2 diabetes.

## 4 Methods

### 4.1 Systematic literature research and data curation

A systematic literature search was conducted for studies reporting glimepiride pharmacokinetic data. PubMed was searched using the keywords glimepiride AND pharmacokinetics, and the PKPDAI database (Gonzalez Hernandez et al., 2021) was queried on 2024–08–30. Inclusion criteria focused on clinical trials involving healthy volunteers or patients with T2DM, and studies investigating the effects of renal impairment, hepatic impairment, bodyweight variations, or *CYP2C9* genotypes on glimepiride pharmacokinetics. Studies involving pediatric populations, non-human subjects, or with insufficiently reported pharmacokinetic data were excluded. The systematic review also included *in vitro* studies providing kinetic parameters (particularly *CYP2C9* activity) required for PBPK model development. The literature review process yielded 20 clinical studies for analysis.

Data from these selected studies were systematically curated and uploaded to the open pharmacokinetics database PK-DB (Grzegorzewski et al., 2021). Patient-specific information (e.g., age, sex, comorbidities, dosing regimens, pharmacokinetic profiles) was extracted following established curation protocols (Grzegorzewski et al., 2021). Figure-based pharmacokinetic data were digitized using WebPlotDigitizer (Rohatgi, 2024), while tabular and textual data were reformatted according to standardized guidelines (Grzegorzewski et al., 2021). Curated data encompassed cohort characteristics, individual-level data, intervention details, time-course concentration profiles of glimepiride and its metabolites, and reported pharmacokinetic/pharmacodynamic parameters. This dataset formed the basis for PBPK model development, calibration, and validation, and is publicly accessible via PK-DB to ensure transparency and reproducibility.

### 4.2 Computational model

The PBPK model and tissue-specific submodels were developed using the Systems Biology Markup Language (SBML) (Hucka et al., 2019; Keating et al., 2020). Programming and visualization of the models were performed using the sbmlutils (König, 2024) and cy3sbml (König et al., 2012) libraries. Numerical solutions for the ordinary differential equations (ODEs) underlying the model were computed using sbmlsim (König, 2021), powered by the high-performance SBML simulation engine libRoadRunner (Welsh et al., 2023; Somogyi et al., 2015).

The developed model comprises a whole-body framework with submodels for the intestine, liver, and kidney to characterize glimepiride’s ADME processes. Key processes include oral dissolution and first-order absorption in the intestine, CYP2C9-mediated hepatic metabolism of glimepiride to M1 followed by further metabolism to M2, and renal excretion of M1 and M2. The mathematical descriptions and ODEs for all submodels are provided in Supplementary Equations S1.1–S1.3. The model and all associated materials (simulation scripts, parameters, and documentation) are publicly available in SBML format under a CC-BY 4.0 license at https://github.com/matthiaskoenig/glimepiride-model, version 0.6.1 (Elias and König, 2025a).

The model was designed to incorporate several key factors influencing inter-individual pharmacokinetic variability. Renal impairment was addressed using the parameter f_renal_function_ (1.0 for normal function), with scaling factors for mild (0.69), moderate (0.32), and severe (0.19) impairment derived from KDIGO guidelines (Stevens et al., 2024) and the approach of (Mallol et al., 2023). This parameter directly scales M1 and M2 metabolite renal excretion rates. Hepatic impairment was implemented via the f_cirrhosis_ parameter (ranging from 0.0 for normal function to 1.0 for severe impairment), with values mapped to the Child-Turcotte-Pugh (CTP) classification (Child and Turcotte, 1964; Infante-Rivard et al., 1987; Köller et al., 2021). This parameter modifies the fraction of functional liver parenchyma and the extent of blood shunting around the liver. Tissue distribution of glimepiride and its metabolites was described via the parameters ftissue_gli_ (rate of tissue distribution) and Kp_gli_ (tissue-plasma partition coefficient), assuming similar distribution properties for the parent drug and metabolites to reduce model complexity. Bodyweight effects were incorporated by scaling organ volumes, blood flows, and metabolic rates according to allometric relationships. *CYP2C9* genetic variability was modeled based on allele-specific scaling factors for the common alleles *1 (wild-type, activity 1.0), *2 (activity 0.68), and *3 (activity 0.23), derived from *in vitro* data (Yang et al., 2018; Maekawa et al., 2009; Dai et al., 2014). Genotype-specific activities were calculated as the mean of the two constituent allele activities. These genetic factors were implemented via the parameter f_cyp2c9_, which modulates the maximal velocity $\left(V_{\text{max}}\right)$ of glimepiride conversion to M1. The Michaelis constant (GLI2M1_Km_gli_) was parameterized using literature values (Suzuki et al., 2006; Maekawa et al., 2009; Zhang et al., 2023). For population-level simulations, observed intrinsic clearance (CL_int_) distribution for diclofenac (a CYP2C9 substrate) (Yang et al., 2012) was characterized using a lognormal function. This distribution shape was retained for modeling allele-specific effects, with the scale parameter adjusted to match the mean activity of each allele. Diplotype activities were calculated as the average contribution of both alleles. Simulations also incorporated published *CYP2C9* genotype frequencies across nine biogeographical populations (ClinPGx, 2025). The Physiome Journal (Elias and König, 2025b) has demonstrated the reproducibility, reusability, and discoverability of the mathematical model and computational simulations.

### 4.3 Model parameterization

Key model parameters related to glimepiride’s absorption, distribution, metabolism, and excretion were optimized by minimizing a weighted sum of squared residuals between model predictions and a curated dataset from clinical studies in healthy, fasted subjects. This optimization utilized multiple (n = 100) runs of a local optimization algorithm. The cost function incorporated weights accounting for study size and measurement variance, ensuring larger studies and more precise measurements had appropriate influence on the optimization. The model was optimized using a subset of the curated clinical data (healthy and fasted), achieving successful convergence and demonstrating good predictive performance across the datasets (see Supplementary Figure S2). The optimized model successfully captured glimepiride pharmacokinetics with satisfactory goodness-of-fit, though some inter-study variability was observed, likely reflecting differences in study design and population characteristics. Final optimized parameters are provided in the Supplementary Table S1. The final parameter set (Supplementary Table S1) was used consistently across all simulations in this study without refitting for each study. Following parameterization, the model’s predictive performance was evaluated across diverse physiological and pathological conditions.

### 4.4 Pharmacokinetic parameters

Standard pharmacokinetic parameters (C_max_, T_max_, AUC, half-life, Cl/F) were calculated from simulated and observed concentration-time profiles using non-compartmental analysis with trapezoidal integration and terminal phase extrapolation. Simulated profiles and derived PK parameters were then compared against the curated experimental data from all 20 clinical studies.

### 4.5 Web application development

To enable real-time simulations, we deployed the glimepiride digital twin as a web application using the Marimo framework. The interface allows users to adjust clinically relevant parameters such as dose, bodyweight, renal and hepatic function, and *CYP2C9* genotype, which are incorporated into the model through the corresponding scaling factors (f_renal_function_, f_cirrhosis_, f_cyp2c9_). Pharmacokinetic parameters (C_max_, T_max_, AUC, half-life) are calculated and displayed alongside the concentration–time profiles. The interface is designed for intuitive use with immediate visual feedback and includes pre-configured example patients for demonstration purposes. The web application is available at https://glimepiride.de, with the code available at https://github.com/matthiaskoenig/glimepiride-app.

## Data Availability

The datasets presented in this study can be found in online repositories. The names of the repository/repositories and accession number(s) can be found below: All curated pharmacokinetic data are publicly available in the PK-DB database (https://pk-db.com) with unique study identifiers.
