# Supplementary material for: A digital twin of glimepiride for personalized and stratified diabetes treatment
Source: Front Pharmacol. 2025 Oct 8;16:1686415. doi: 10.3389/fphar.2025.1686415 (PMC12540372; doi:10.3389/fphar.2025.1686415)
Supplement: Supplementary file 1 [file DataSheet1.pdf]

Supplementary Material

CONTENTS

S1 Prisma Workflow (Fig. S1) 2
S2 Submodels (Fig. S2) 3
S3 Model Equations 4
S4 Parameter Optimization 10
S4.1 Optimal Parameters (Tab. S1) 10
S4.2 Parameter Optimization Results (Fig. S3) 10
S5 Simulations 11
S5.1 Dose Dependency (Fig. S4) 11
S5.2 Renal Impairment (Fig. S5) 12
S5.3 Hepatic Impairment (Fig. S6) 13
S5.4 Bodyweight Dependency (Fig. S7) 14
S5.5 CYP2C9 Polymorphisms (Fig. S8) 15
S6 Study Simulations 16
S6.1 Ahmed2016 (Fig. S9) 16
S6.2 Badian1994 (Fig. S10 – S11) 16
S6.3 Badian1996 (Fig. S12) 17
S6.4 Choi2014 (Fig. S13) 18
S6.5 FDA (Fig. S14 – S16) 18
S6.6 Helmy2013 (Fig. S17) 20
S6.7 Kasichayanula2011c (Fig. S18) 20
S6.8 Kim2017 (Fig. S19) 21
S6.9 Lee2012 (Fig. S20) 21
S6.10 Lehr1990 (Fig. S21) 22
S6.11 Liu2010 (Fig. S22) 22
S6.12 Malerczyk1994 (Fig. S23) 23
S6.13 Matsuki2007 (Fig. S24) 23
S6.14 Niemi2002 (Fig. S25) 24
S6.15 Ratheiser1993 (Fig. S26) 24
S6.16 Rosenkranz1996a (Fig. S27 – S28) 25
S6.17 Shukla2004 (Fig. S29 – S30) 26
S6.18 Suzuki2006 (Fig. S31) 27
S6.19 Wang2005 (Fig. S32) 27
S6.20 Yoo2011 (Fig. S33) 28
S7 CYP2C9 Analysis 28
S7.1 Activity of CYP2C9 Alleles (Tab. S2) 28
S7.2 CYP2C9 Intrinsic Clearance (Tab. S3) 29
S7.3 CYP2C9 Lognormal Distribution (Tab. S4) 29
S7.4 Sampled CYP2C9 Allele Activities (Tab. S5) 30
S7.5 Sampled Glimepiride PK Parameters by CYP2C9 Genotype (Tab. S6) 30
S7.6 Sampled M1 PK Parameters by CYP2C9 Genotype (Tab. S7) 31
S7.7 Sampled M2 PK Parameters by CYP2C9 Genotype (Tab. S8) 31
S8 Biogeographical Groups Analysis 32
S8.1 AUC by Biogeographical Groups (Tab. S9) 32
S8.2 T_{max} by Biogeographical Groups (Tab. S10) 32

S8.3 C_{max} by Biogeographical Groups (Tab. S11) 33
S8.4 Most Significant Differences by Biogeographical Groups (Tab. S12) 33
S8.5 Sampled CYP2C9 Genotypes Freqiencies by Biogeographical Groups (Tab. S13) 34

S1 PRISMA WORKFLOW (FIG. S1)

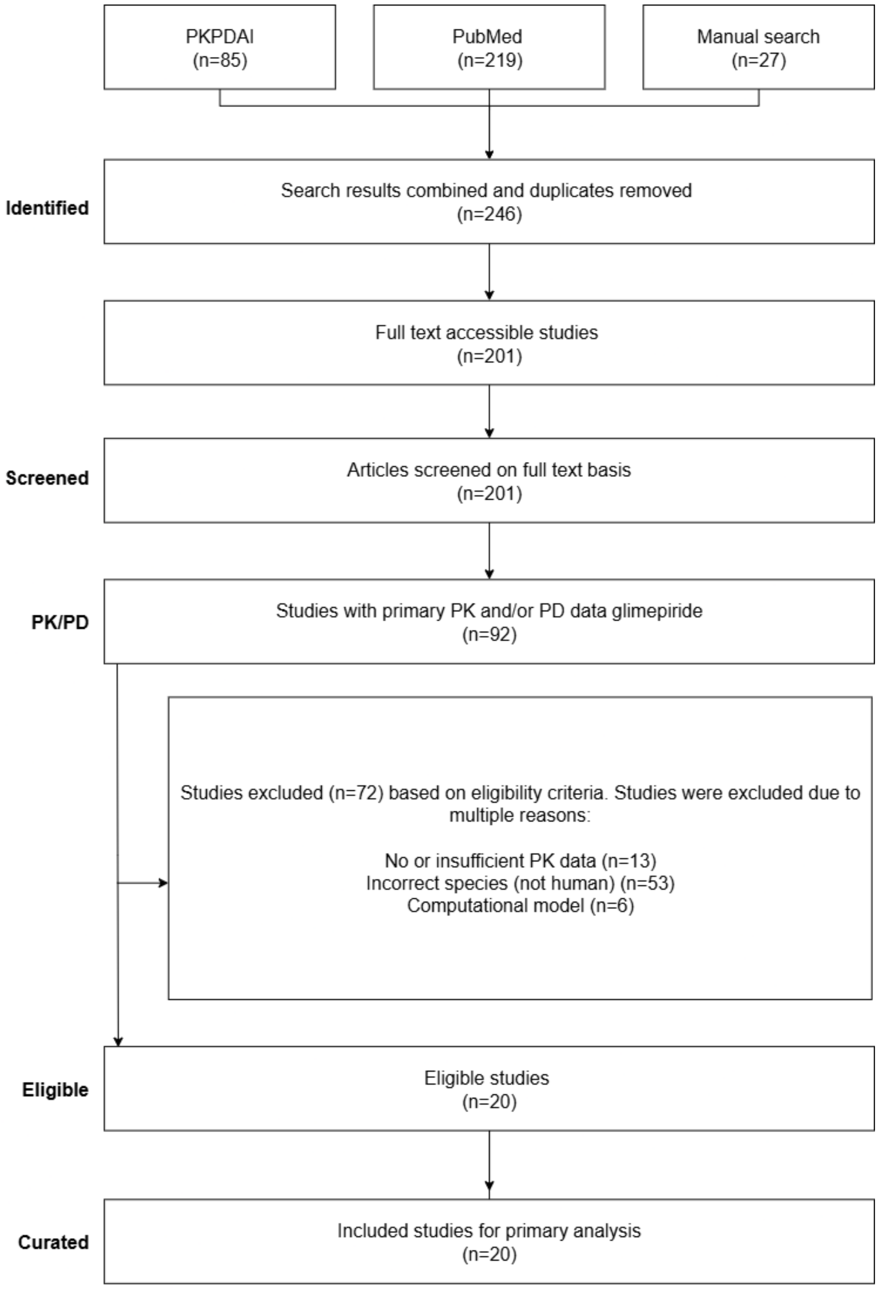

Figure S1: **PRISMA flow diagram.** Overview of data selection for the pharmacokinetics dataset of glimepiride established in this work. PubMed, PKPDAI, and manual searches were used for the literature search on the pharmacokinetics of glimepiride. Application of the eligibility criteria resulted in 19 studies.

S2 SUBMODELS (FIG. S2)

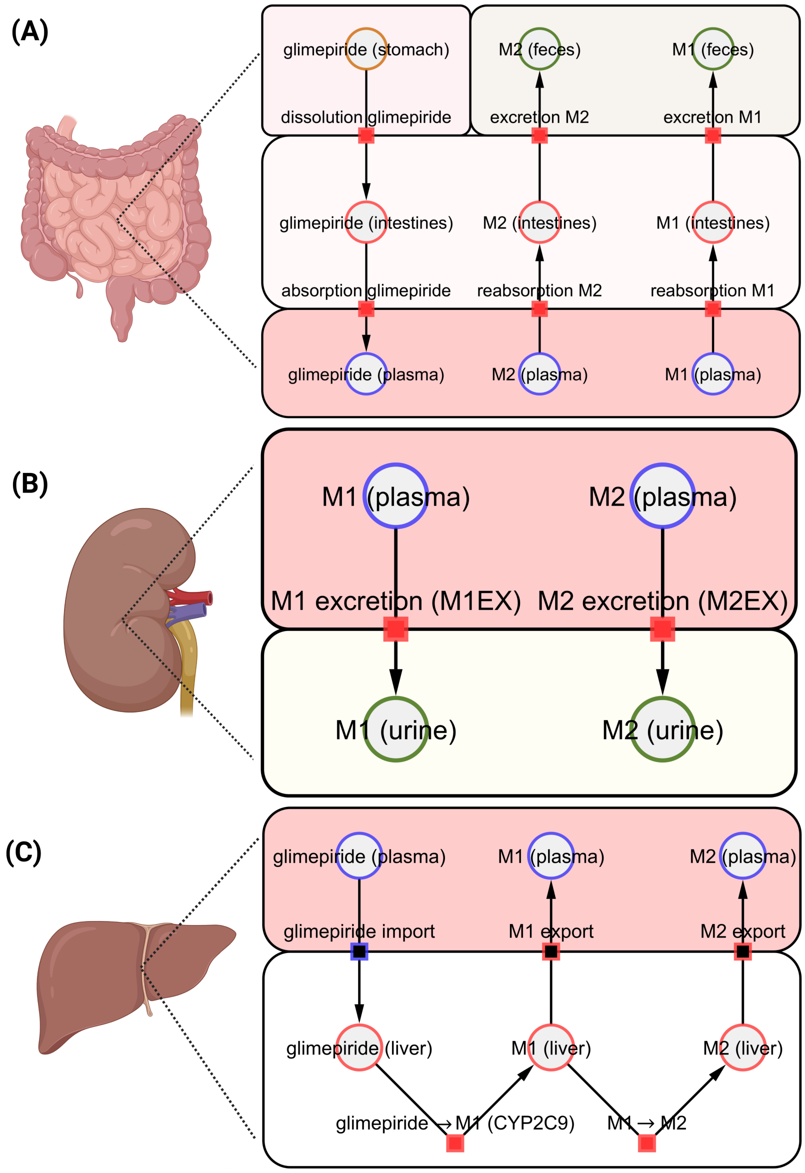

Figure S2: **Visualizations of the submodels. (A) Intestine model:** Glimepiride dissolves in the stomach, then transfers to the intestinal lumen, where it can be absorbed into plasma. Metabolites M1 and M2 undergo reabsorption into the intestinal lumen and excretion into feces. **(B) Kidney model:** Metabolites M1 and M2 are transported from plasma to urine. **(C) Liver model:** Glimepiride is transported from plasma to the liver, where it undergoes CYP2C9-mediated metabolism to form M1. This intermediate is further converted to M2. Both M1 and M2 are then exported back to plasma.

S3 MODEL EQUATIONS

The model and all associated materials (simulation scripts, parameters, and documentation) are publicly available in SBML format under a CC-BY 4.0 license at `https://github.com/matthiaskoenig/glimepiride-model`, version 0.6.1 Elias and König (2025), which also contains detailed model reports. The following sections provide an overview of the mathematical descriptions and ODEs for all submodels.

Intestine Model

Dissolution of glimepiride ($\mathrm{PODOSE}_{\mathrm{gli}}$) (mass to amount):

$$\mathrm{dissolution}_{gli} = \frac{\mathrm{Ka}_{dis\_gli}}{60} \cdot \frac{\mathrm{PODOSE}_{gli}}{\mathrm{Mr}_{gli}}$$

where Ka_{dis_gli} [hr^{-1}] is scaled by $\frac{1}{60}$ to min^{-1}, PODOSE_{gli} is the oral dose [mg], and Mr_{gli} [g/mol] is the molecular weight of glimepiride.

The corresponding ODE is:

$$\frac{d\,PODOSE_{\mathrm{gli}}}{dt} = -\mathrm{dissolution}_{gli} \cdot \mathrm{Mr}_{gli}$$

Absorption rate from intestinal lumen to systemic circulation:

$$\mathrm{GLIABS} = f_{\mathrm{absorption}} \cdot \mathrm{GLIABS}_k \cdot V_{\mathrm{lumen}} \cdot \mathrm{gli}_{lumen}$$

where $f_{\mathrm{absorption}}$ is a scaling factor for food effects, GLIABS_k [min^{-1}] is the first-order absorption rate constant, V_{lumen} [L] is the volume of the intestinal lumen, and gli_{lumen} [mmol/L] is the lumen concentration of glimepiride.

Net change in lumen glimepiride concentration:

$$\frac{d\,\mathrm{gli}_{lumen}}{dt} = -\frac{\mathrm{GLIABS}}{V_{\mathrm{lumen}}} + \frac{\mathrm{dissolution}_{gli}}{V_{\mathrm{lumen}}}$$

Reabsorption rates for M1 (M1REABS) and M2 (M2REABS) from plasma to intestinal lumen:

$$\mathrm{M1REABS} = \mathrm{MREABS}_k \cdot \mathrm{m1}_{ext} \cdot V_{\mathrm{lumen}}$$

$$\mathrm{M2REABS} = \mathrm{MREABS}_k \cdot \mathrm{m2}_{ext} \cdot V_{\mathrm{lumen}}$$

where $\mathrm{m1}_{ext}$ and $\mathrm{m2}_{ext}$ [mmol/L] are the plasma concentrations of M1 and M2, respectively, and MREABS_k [min^{-1}] is the first-order reabsorption rate constant.

Excretion from lumen to feces for M1 (M1EXC) and M2 (M2EXC):

$$\mathrm{M1EXC} = \mathrm{MEXC}_k \cdot \mathrm{m1}_{lumen} \cdot V_{\mathrm{lumen}}$$

$$\mathrm{M2EXC} = MEXC_{\mathrm{k}} \cdot m2_{\mathrm{lumen}} \cdot V_{\mathrm{lumen}}$$

where $\mathrm{m1}_{lumen}$ and $\mathrm{m2}_{lumen}$ [mmol/L] are lumen concentrations of M1 and M2, respectively, and MEXC_k [min^{-1}] is the excretion rate constant.

Net changes in lumen concentrations are:

$$\frac{d\,\mathrm{m1}_{lumen}}{dt} = \frac{\mathrm{M1REABS}}{V_{\mathrm{lumen}}} - \frac{\mathrm{M1EXC}}{V_{\mathrm{lumen}}}$$

$$\frac{d\,\mathrm{m2}_{lumen}}{dt} = \frac{\mathrm{M2REABS}}{V_{\mathrm{lumen}}} - \frac{\mathrm{M2EXC}}{V_{\mathrm{lumen}}}$$

Metabolite accumulation in feces(cumulative amounts $\mathrm{m1}_{feces}$, $\mathrm{m2}_{feces}$ [mmol]):

$$\frac{d\,\mathrm{m1}_{feces}}{dt} = \mathrm{M1EXC}$$

$$\frac{d\,\mathrm{m2}_{feces}}{dt} = \mathrm{M2EXC}$$

Total fecal metabolites:

$$\mathrm{mtot}_{feces} = \mathrm{m1}_{feces} + \mathrm{m2}_{feces}$$

Plasma concentration changes due to absorption/reabsorption are:

$$\frac{d\,\mathrm{gli}_{ext}}{dt} = \frac{\mathrm{GLIABS}}{V_{ext}}$$

where V_{ext} [L] is the plasma volume.

Plasma M1 and M2 concentration changes due to reabsorption from lumen:

$$\frac{d\,\mathrm{m1}_{ext}}{dt} = -\frac{\mathrm{M1REABS}}{V_{ext}}$$

$$\frac{d\,\mathrm{m2}_{ext}}{dt} = -\frac{\mathrm{M2REABS}}{V_{ext}}$$

Key parameters for the intestinal model:

- $\mathrm{Ka}_{\mathrm{dis\_gli}}$ [hr^{-1}]: Dissolution rate constant for glimepiride.
- $\mathrm{GLIABS}_{\mathrm{k}}$ [min^{-1}]: Absorption rate constant.
- $\mathrm{MREABS}_{\mathrm{k}}$, MEXC_{k} [min^{-1}]: Reabsorption and excretion rate constants for M1 and M2.
- V_{lumen} [L]: Volume of the intestinal lumen.
- $\mathrm{Mr}_{\mathrm{gli}}$ [g/mol]: Molecular weight of glimepiride.
- $\mathrm{f}_{\mathrm{absorption}}$ [-]: Scaling factor for food effects.
- $\mathrm{V}_{\mathrm{ext}}$ [L]: Plasma volume.

Kidney Model

Renal excretion rates for M1 (M1EX) and M2 (M2EX):

$$\mathrm{M1EX} = f_{\mathrm{renal\_function}} \cdot V_{\mathrm{ki}} \cdot \mathrm{M1EX}_{k} \cdot m1_{\mathrm{ext}}$$

$$\mathrm{M2EX} = f_{\mathrm{renal\_function}} \cdot V_{\mathrm{ki}} \cdot \mathrm{M2EX}_{k} \cdot m2_{\mathrm{ext}}$$

where $f_{\mathrm{renal\_function}}$ is a scaling factor, V_{ki} [L] is the kidney compartment volume, $\mathrm{M1EX}_{k}$ and $\mathrm{M2EX}_{k}$ [min^{-1}] are excretion rate constants, and $m1_{\mathrm{ext}}$, $m2_{\mathrm{ext}}$ [mmol/L] are the plasma concentrations.

Estimated glomerular filtration rate (eGFR):

$$\mathrm{egfr} = f_{\mathrm{renal\_function}} \cdot \mathrm{egfr}_{healthy}$$

where $\text{egfr}_{\text{healthy}}$ [ml/min/m^2] represents the typical eGFR value in a healthy individual.

Creatinine clearance (crcl) derivation:

$$\text{crcl} = \frac{\text{egfr} \cdot \text{BSA}}{1.73} \cdot 1.1$$

where BSA [m^2] is the body surface area, crcl is expressed in [mL/min], 1.73 [m^2] is the standard adult BSA used for normalization, and the factor 1.1 is a correction factor that accounts for the systematic overestimation of creatinine clearance compared to the actual GFR.

ODEs for plasma concentrations and urine amounts:

For M1:

$$\frac{d\,m1_{\text{ext}}}{dt} = -\frac{\text{M1EX}}{V_{\text{ext}}}$$
$$\frac{d\,m1_{\text{urine}}}{dt} = \text{M1EX}$$

For M2:

$$\frac{d\,m2_{\text{ext}}}{dt} = -\frac{\text{M2EX}}{V_{\text{ext}}}$$
$$\frac{d\,m2_{\text{urine}}}{dt} = \text{M2EX}$$

where V_{ext} [L] is the plasma volume.

Key parameters for the kidney model:

- M1EX_k, M2EX_k [min^{-1}]: First-order excretion rate constants for M1 and M2.
- V_{ki} [L]: Kidney compartment volume.
- V_{ext} [L]: Plasma volume in the kidney.
- $f_{\text{renal\_function}}$ [-]: Scaling factor to account for normal or impaired renal function.

Liver Model

Glimepiride import rate from plasma to liver (GLIIM):

$$\text{GLIIM} = \text{GLIIM}_k \cdot V_{\text{li}} \cdot \left(\text{gli}_{ext} - \text{gli}\right)$$

where GLIIM_k [min^{-1}] is the import rate constant, V_{li} [L] is the liver volume, and gli_{ext}, gli [mmol/L] are the glimepiride concentrations in plasma and liver, respectively.

Glimepiride conversion to M1:

$$\text{GLI2M1} = f_{\text{cyp2c9}} \cdot \text{GLI2M1}_{Vmax} \cdot V_{\text{li}} \cdot \frac{\text{gli}}{\text{gli} + \text{GLI2M1}_{Km\_gli}}$$

where f_{cyp2c9} is a scaling factor for CYP2C9 activity, GLI2M1_{Vmax} [$\text{mmol}\,\text{min}^{-1}\,\text{L}^{-1}$] is the maximum rate of conversion of GLI to M1, and GLI2M1_Km_gli [mmol/L] is the Michaelis constant.

M1 export to plasma (M1EX):

$$\text{M1EX} = \text{M1EX}_k \cdot V_{\text{li}} \cdot \left(\text{m1} - \text{m1}_{ext}\right)$$

M1 conversion to M2 (M12M2):

$$\text{M12M2} = \text{M12M2}_k \cdot V_{\text{li}} \cdot \text{m1}$$

where M12M2_k [min^{-1}] is the rate constant, and m1 [mmol/L] is the M1 concentration in the liver.

M2 export rate to plasma (M2EX):

$$\text{M2EX} = \text{M2EX}_k \cdot V_{\text{li}} \cdot \left(\text{m2} - \text{m2}_{ext}\right)$$

where m2 and m2_{ext} are liver and plasma concentration, and M2EX_k [min^{-1}] is the M2 export rate constant.

ODEs for liver concentrations:

$$\begin{aligned}
\frac{d\,\text{gli}}{dt} &= \frac{\text{GLIIM}}{V_{\text{li}}} - \frac{\text{GLI2M1}}{V_{\text{li}}} \\
\frac{d\,\text{m1}}{dt} &= \frac{\text{GLI2M1}}{V_{\text{li}}} - \frac{\text{M1EX}}{V_{\text{li}}} - \frac{\text{M12M2}}{V_{\text{li}}} \\
\frac{d\,\text{m2}}{dt} &= \frac{\text{M12M2}}{V_{\text{li}}} - \frac{\text{M2EX}}{V_{\text{li}}}
\end{aligned}$$

ODEs for plasma concentration changes due to liver exchange/metabolism:

$$\frac{d\,\mathrm{gli}_{\mathrm{ext}}}{dt} = -\frac{\mathrm{GLIIM}}{V_{\mathrm{ext}}}$$
$$\frac{d\,\mathrm{m1}_{\mathrm{ext}}}{dt} = \frac{\mathrm{M1EX}}{V_{\mathrm{ext}}}$$
$$\frac{d\,\mathrm{m2}_{\mathrm{ext}}}{dt} = \frac{\mathrm{M2EX}}{V_{\mathrm{ext}}}$$

This model assumes glimepiride is fully metabolized by the liver. CYP2C9 enzyme activity is varied by f_{cyp2c9}. Glimepiride to M1 conversion follows Michaelis-Menten kinetics, while M1 to M2 conversion follows mass-action kinetics. Metabolite exports are modeled as first-order processes driven by concentration gradients.

Key parameters for the liver model:

- GLIIM_k [min^{-1}]: Glimepiride import rate constant (plasma → liver).
- $\mathrm{GLI2M1}_{Vmax}$ [$\mathrm{mmol\,min^{-1}\,L^{-1}}$], GLI2M1_Km_gli [mmol/L]: Michaelis-Menten parameters for glimepiride → M1 conversion.
- $\mathrm{M12M2}_k$ [min^{-1}]: First-order rate constant for M1 → M2.
- $\mathrm{M1EX}_k, \mathrm{M2EX}_k$ [min^{-1}]: Rate constants for M1 and M2 export.
- f_{cyp2c9} [-]: Scaling factor for CYP2C9 activity.
- V_{li}, V_{ext} [L]: Volumes of liver and plasma compartments, respectively.

S4 PARAMETER OPTIMIZATION

S4.1 Optimal Parameters (Tab. S1)

Table S1. Optimized parameters for the glimepiride PBPK model.

Parameter	Value	Unit	Description
GU__GLIABS$_k$	0.01590	min^{-1}	Absorption rate constant of glimepiride into plasma.
GU__MREABS$_k$	0.01592	min^{-1}	Reabsorption rate constant of metabolites into the intestines.
GU__MEXC$_k$	0.00017	min^{-1}	Fecal excretion rate constant of metabolites.
LI__GLI2M1$_{Vmax}$	0.00005	mmole/min/L	Maximum velocity of glimepiride to M1 conversion in the liver.
LI__M1EX$_k$	0.07774	min^{-1}	Transport rate constant of M1 from the liver to plasma.
LI__M12M2$_k$	0.01485	min^{-1}	Rate constant of M1 to M2 conversion in the liver.
LI__M2EX$_k$[1]	99.99817	min^{-1}	Transport rate constant of M2 from the liver to plasma.
KI__M1EX$_k$	0.14801	min^{-1}	Renal excretion rate constant of M1 into urine.
KI__M2EX$_k$	0.09849	min^{-1}	Renal excretion rate constant of M2 into urine.
ftissue$_{gli}$	0.00071	L/min	Tissue-to-plasma partition coefficient of glimepiride.
Kp$_{gli}$[2]	10.02060	-	Partition coefficient for glimepiride distribution.

A total of 100 optimization runs were performed.
[1] Reached upper bound during parameter optimization.
[2] Reached lower bound during parameter optimization.

S4.2 Parameter Optimization Results (Fig. S3)

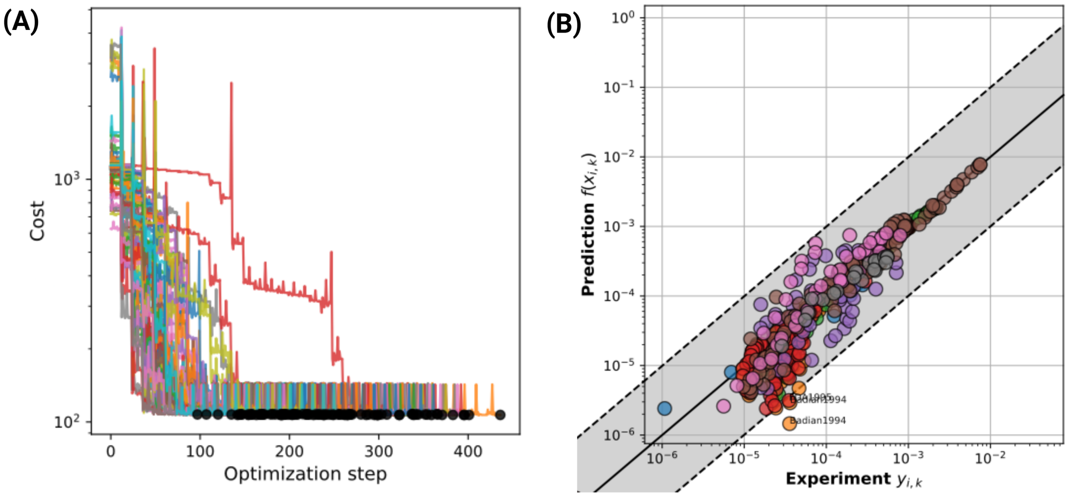

Figure S3: **Results of the parameter fitting. a)** Cost reduction over optimization steps. **b)** Goodness-of-fit plot comparing model predictions to experimental data.

S5 SIMULATIONS

S5.1 Dose Dependency (Fig. S4)

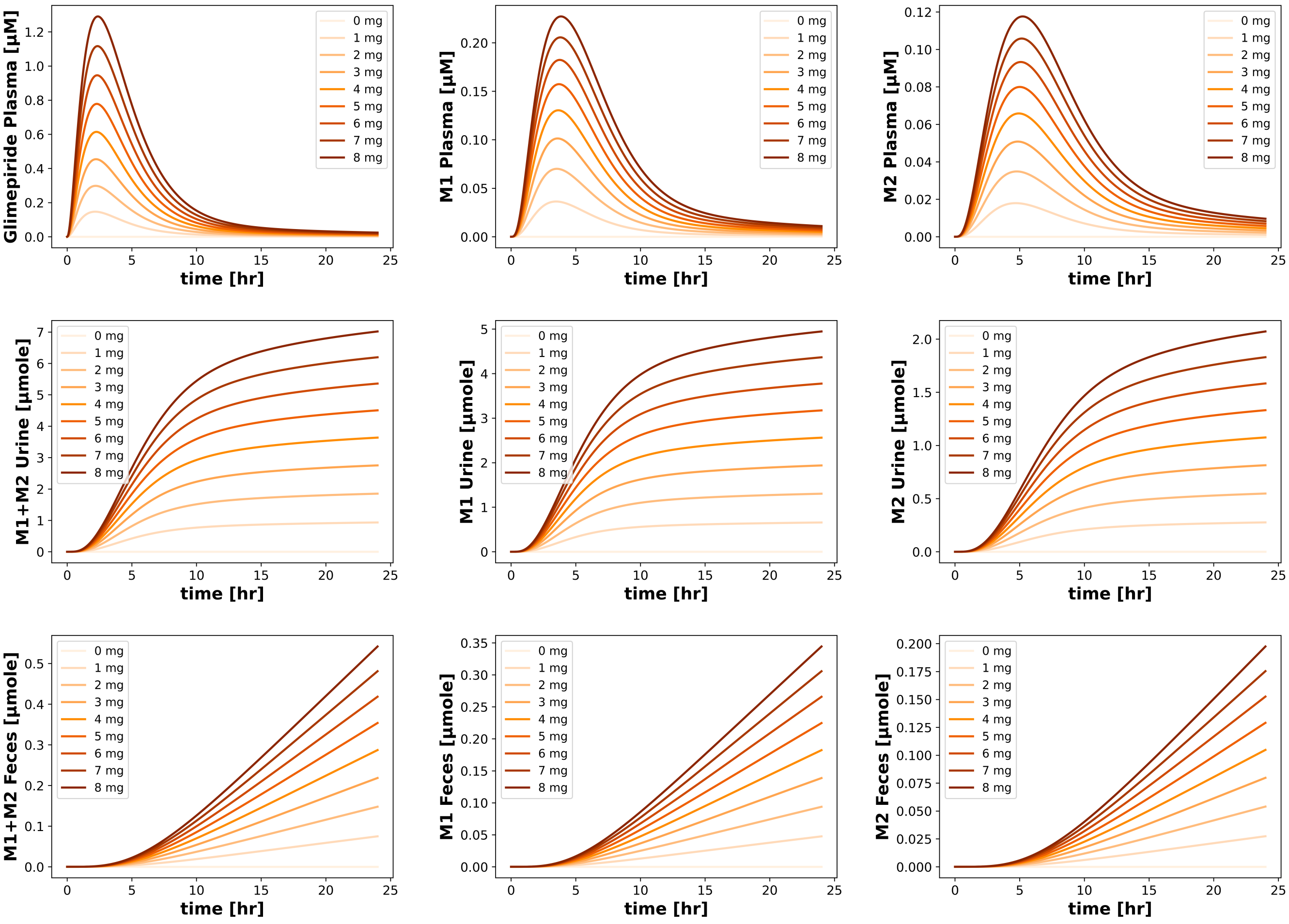

Figure S4: **Simulated concentration-time and cumulative excretion-time profiles of glimepiride and its primary metabolites following various oral doses.** Profiles were generated for multiple glimepiride doses to demonstrate dose dependency in absorption, metabolism, and excretion.

S5.2 Renal Impairment (Fig. S5)

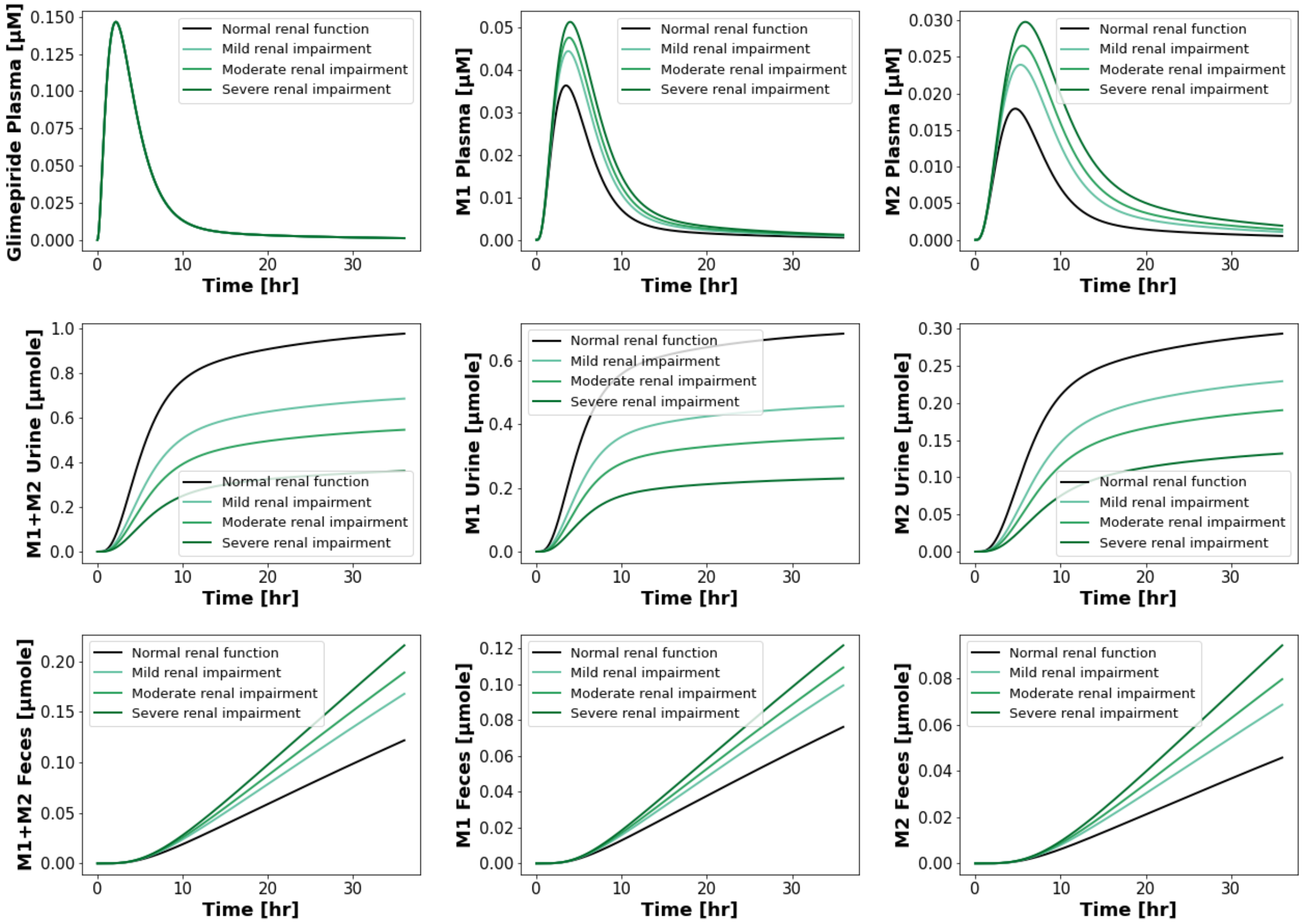

Figure S5: **Simulated pharmacokinetic profiles of glimepiride (3 mg dose) and its metabolites across varying degrees of renal impairment.** Concentration-time curves and cumulative excretion patterns are compared to normal renal function.

S5.3 Hepatic Impairment (Fig. S6)

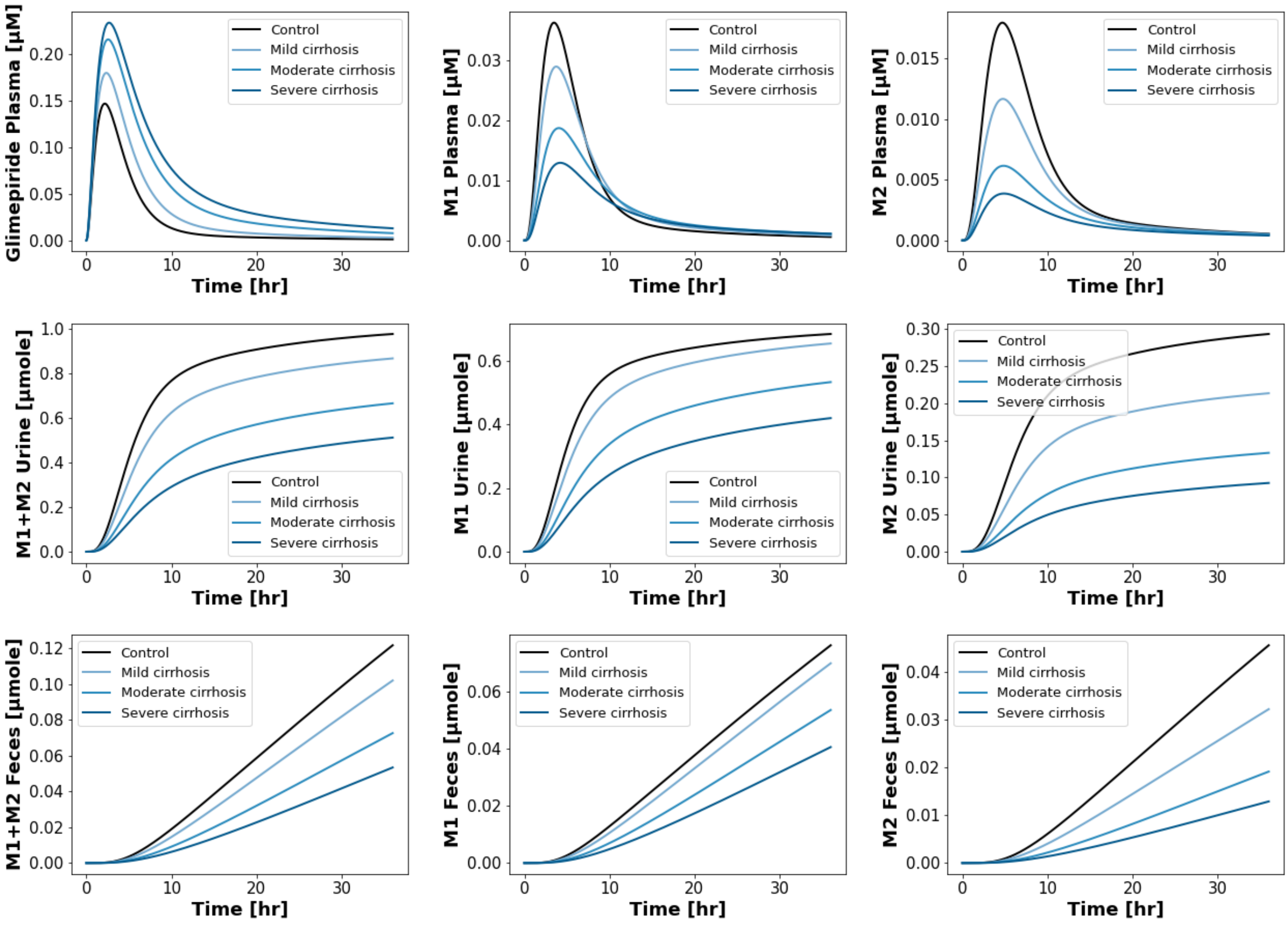

Figure S6: **Simulated pharmacokinetic profiles of glimepiride (1 mg dose) and its metabolites across varying degrees of cirrhosis.** Concentration-time curves and cumulative excretion patterns are compared to normal (control) hepatic function.

S5.4 Bodyweight Dependency (Fig. S7)

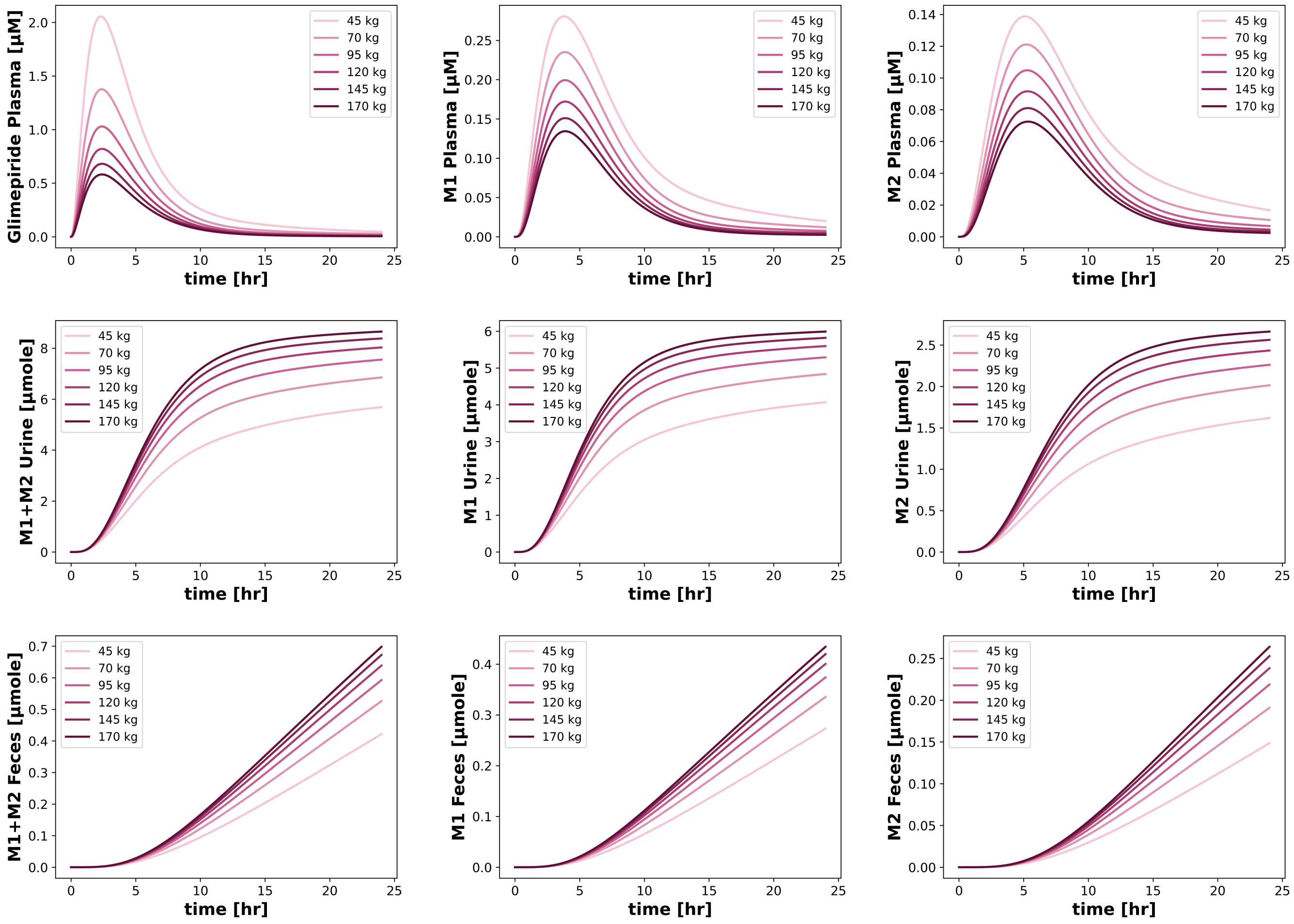

Figure S7: **Simulated pharmacokinetic profiles of glimepiride (8 mg dose) and its metabolites across different bodyweights.** Concentration-time curves and cumulative excretion patterns illustrate the impact of bodyweight on drug disposition.

S5.5 CYP2C9 Polymorphisms (Fig. S8)

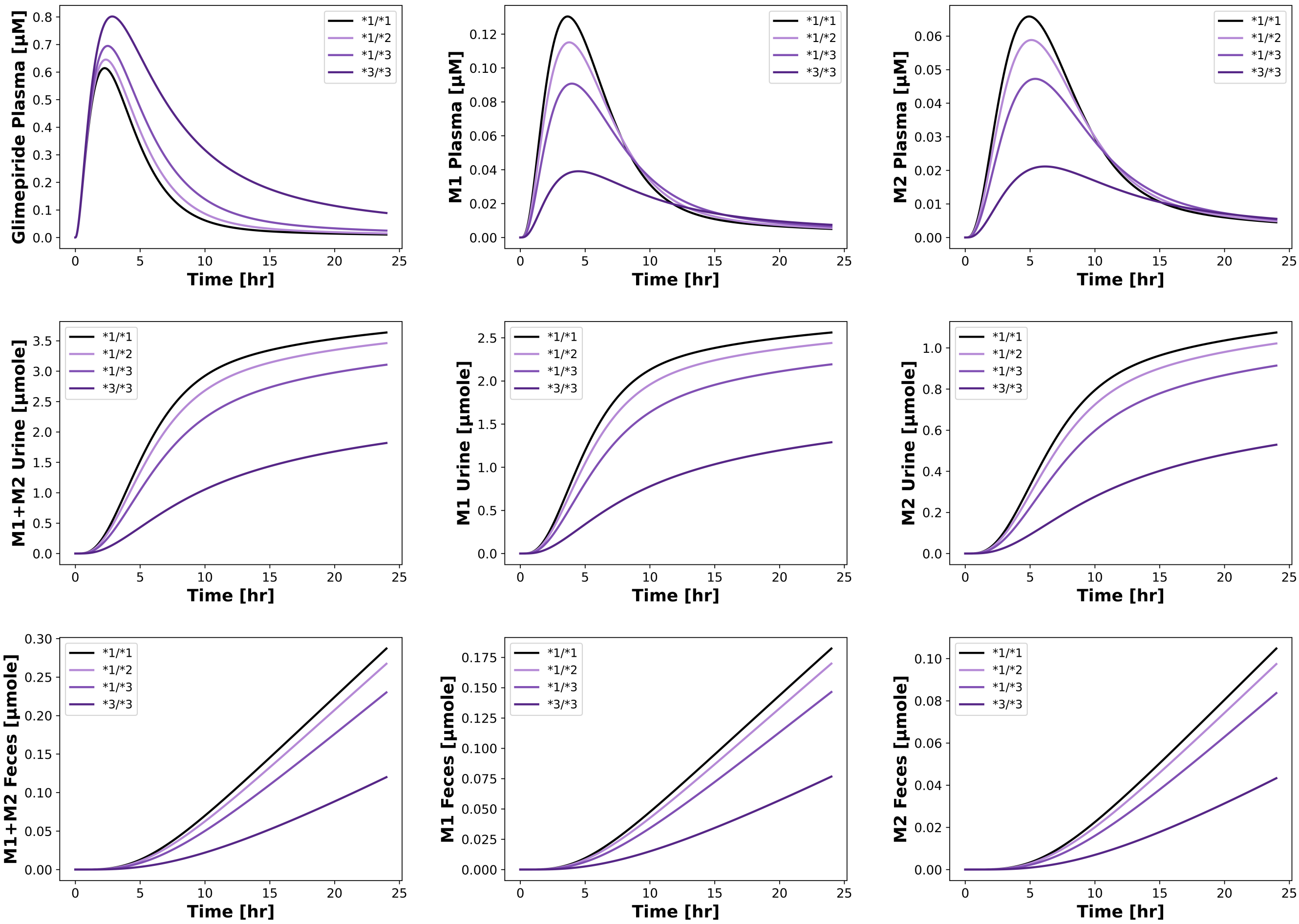

Figure S8: **Simulated pharmacokinetic profiles of glimepiride (4 mg dose) and its metabolites across the main CYP2C9 genotypes.** Concentration-time curves and cumulative excretion patterns demonstrate the impact of genetic polymorphisms on drug metabolism.

S6 STUDY SIMULATIONS

S6.1 Ahmed2016 (Fig. S9)

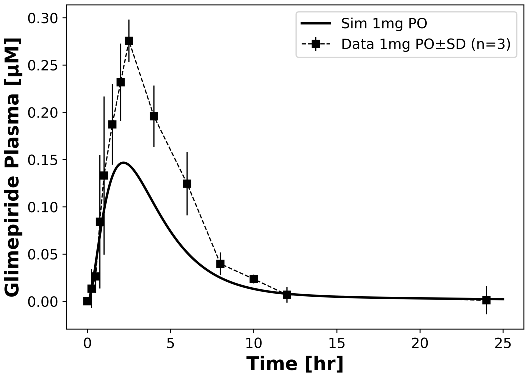

Figure S9: **Simulation Ahmed2016 Ahmed et al. (2016).** Simulated and observed glimepiride plasma concentrations after a 1 mg oral dose in healthy Egyptian volunteers. Error bars represent ±SD (n=3).

S6.2 Badian1994 (Fig. S10 – S11)

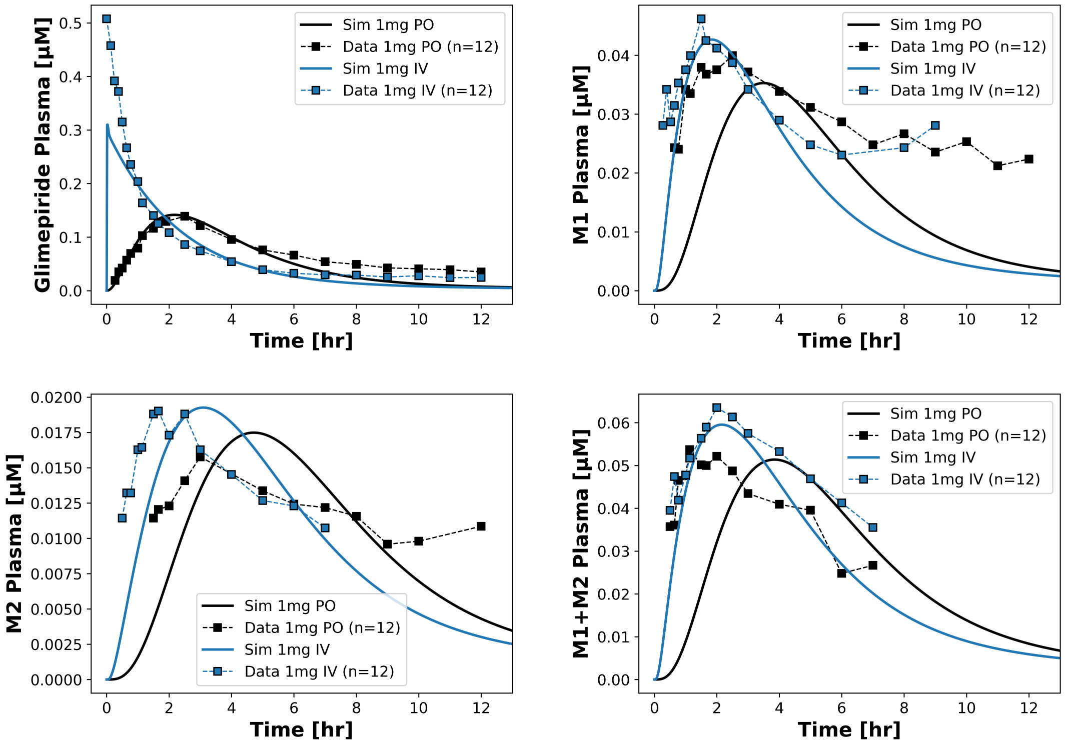

Figure S10: **Simulation Badian1994 Badian et al. (1994).** Simulated and observed plasma concentrations of glimepiride, M1, M2, and their combined total (M1+M2) following a 1 mg (oral or IV) dose in healthy Caucasian (n=12) volunteers.

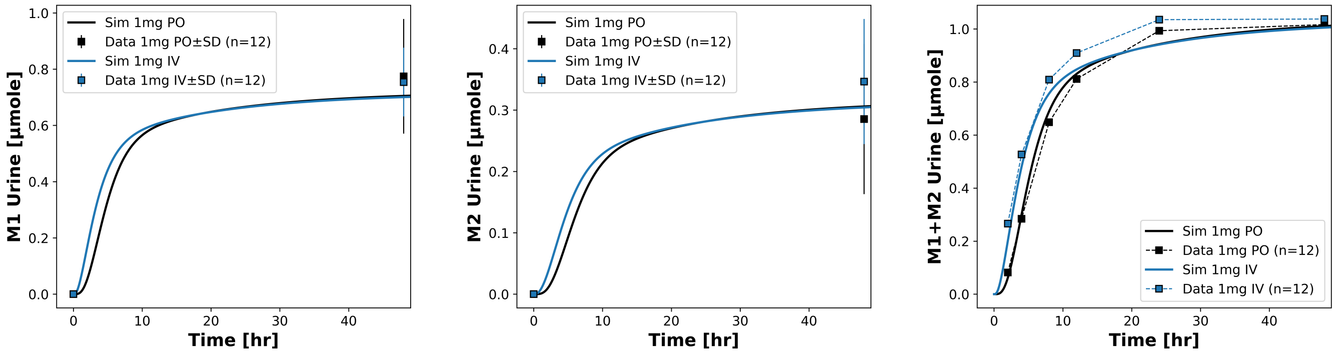

Figure S11: **Simulation Badian1994 Badian et al. (1994).** Simulated and observed urinary excretion of M1, M2, and their total (M1+M2), following a 1 mg (oral or IV) dose in healthy Caucasian (n=12) volunteers.

S6.3 Badian1996 (Fig. S12)

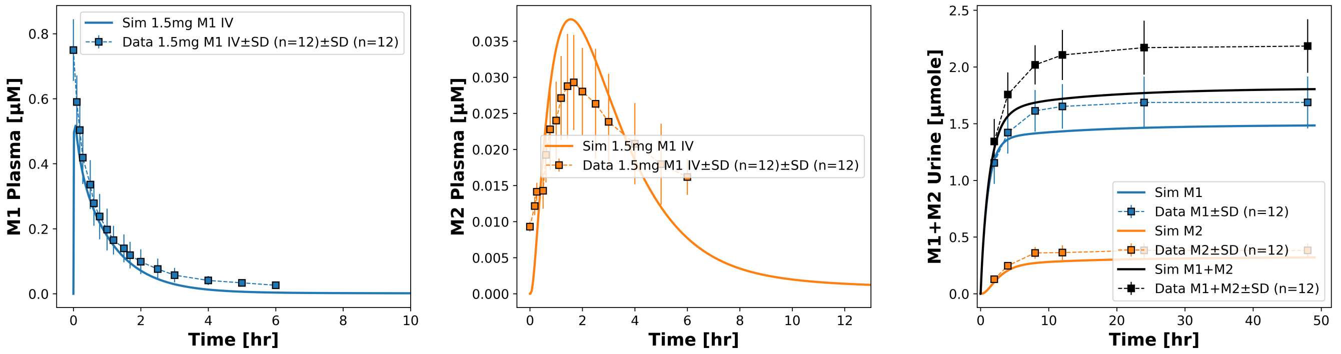

Figure S12: **Simulation Badian1996 Badian et al. (1996).** Simulated and observed plasma concentrations of M1 and M2, as well as urinary excretion of their combined total (M1+M2), following a 1.5 mg intravenous dose in healthy Caucasian volunteers. Error bars indicate ±SD (n=12).

S6.4 Choi2014 (Fig. S13)

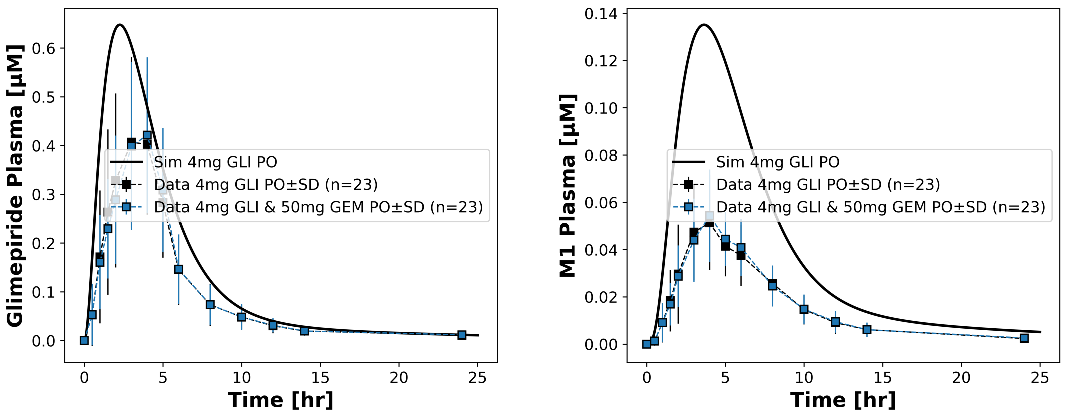

Figure S13: **Simulation Choi2014 Choi et al. (2014).** Simulated and observed glimepiride and M1 plasma concentrations following a 4 mg oral dose of glimepiride, alone or in combination with 50 mg gemigliptin, in healthy volunteers. Error bars represent ±SD (n=23).

S6.5 FDA (Fig. S14 – S16)

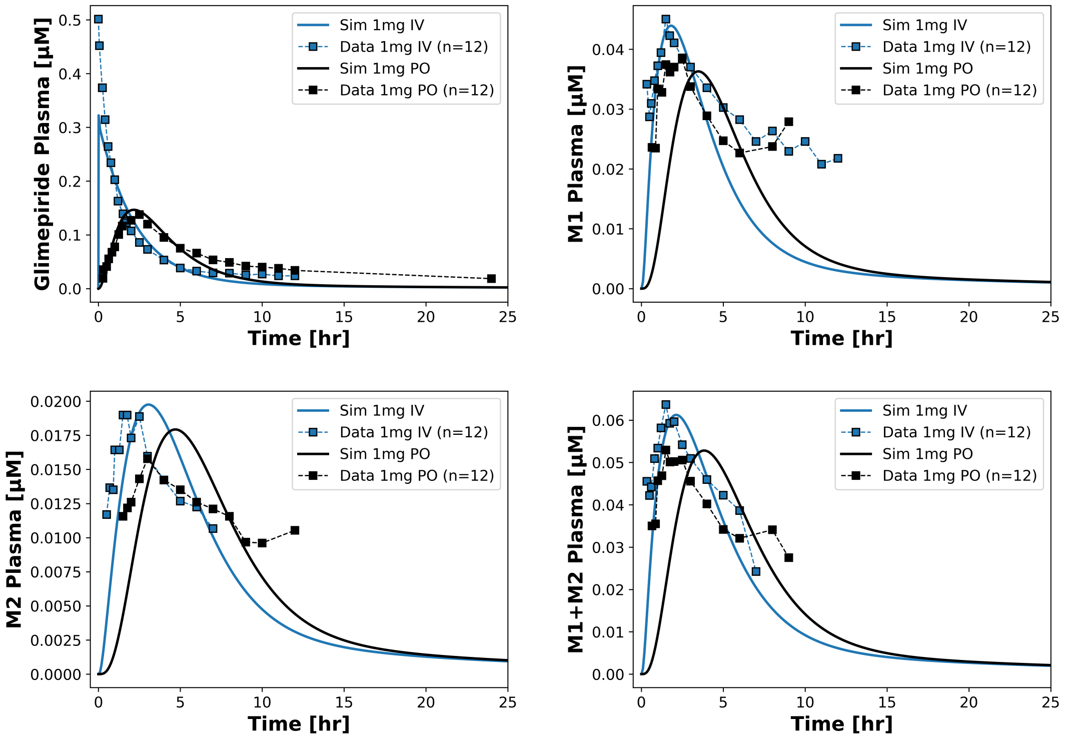

Figure S14: **Simulation FDA U.S. Food and Drug Administration (FDA) (1995).** Simulated and observed plasma concentrations of glimepiride, M1, M2, and their combined amounts (M1 + M2) after a 1 mg oral or intravenous dose in healthy Korean (n=12) volunteers.

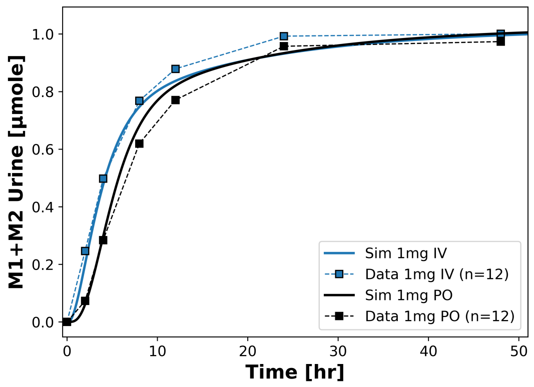

Figure S15: **Simulation FDA U.S. Food and Drug Administration (FDA) (1995).** Simulated and observed cumulative urinary metabolite (M1 + M2) excretion after a 1 mg glimepiride intravenous or oral dose in healthy (n=12) volunteers.

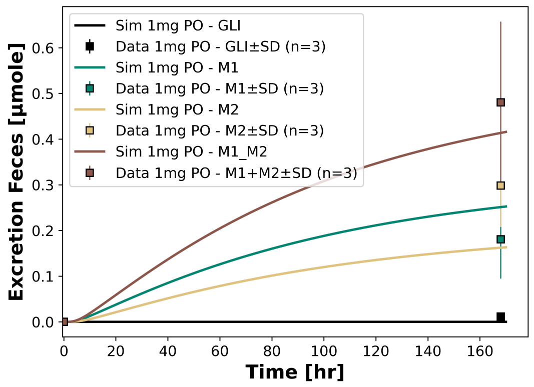

Figure S16: **Simulation FDA U.S. Food and Drug Administration (FDA) (1995).** Simulated versus observed cumulative fecal excretion of glimepiride, M1, M2, and total metabolites (M1 + M2) after a 1 mg oral dose in healthy (n=3) volunteers.

S6.6 Helmy2013 (Fig. S17)

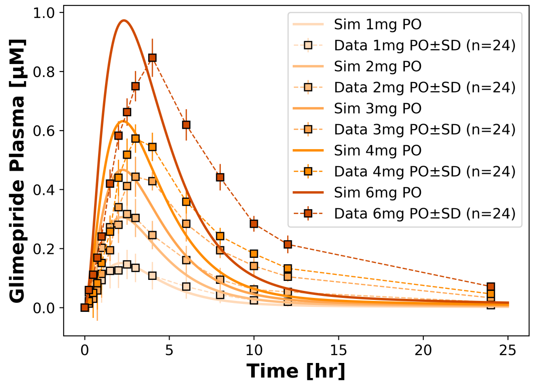

Figure S17: **Simulation Helmy2013 Helmy et al. (2013).** Simulated versus observed glimepiride plasma concentrations after oral doses of 1–6 mg in healthy Egyptian volunteers (n=24).

S6.7 Kasichayanula2011c (Fig. S18)

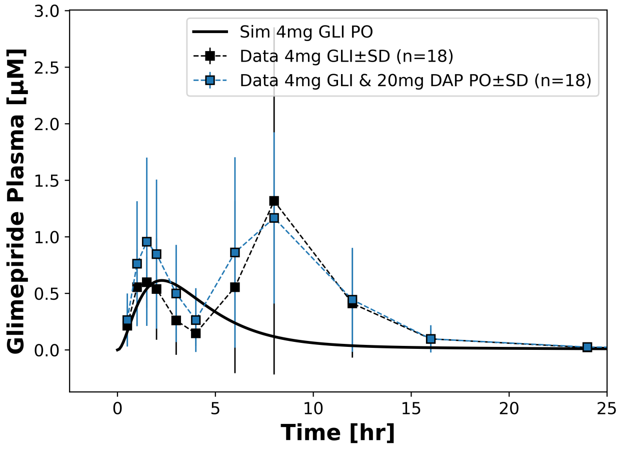

Figure S18: **Simulation Kasichayanula2011c Kasichayanula et al. (2011).** Simulated versus observed glimepiride plasma concentrations after a 4 mg oral dose in healthy volunteers, with or without co-administration of 20 mg dapagliflozin. Error bars represent ±SD (n=18). Note: This study was classified as an outlier during model development due to the atypical double-peak concentration profile.

S6.8 Kim2017 (Fig. S19)

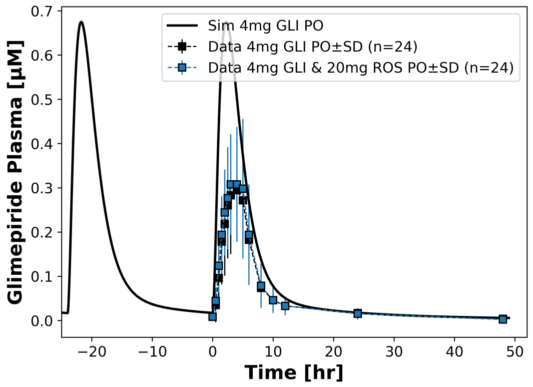

Figure S19: **Simulation Kim2017 Kim et al. (2017).** Simulated versus observed glimepiride plasma concentrations after multiple 4 mg oral doses in healthy Korean volunteers, with and without co-administration of 20 mg rosuvastatin. Error bars represent ±SD (n=24).

S6.9 Lee2012 (Fig. S20)

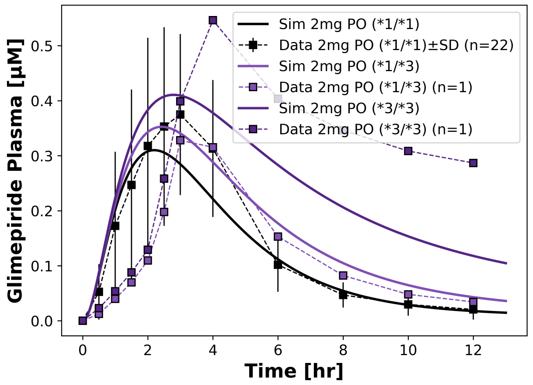

Figure S20: **Simulation Lee2012 Lee et al. (2012).** Simulated versus observed glimepiride plasma concentrations after a 2 mg oral dose in healthy Korean volunteers. Error bars represent ±SD (n=24).

S6.10 Lehr1990 (Fig. S21)

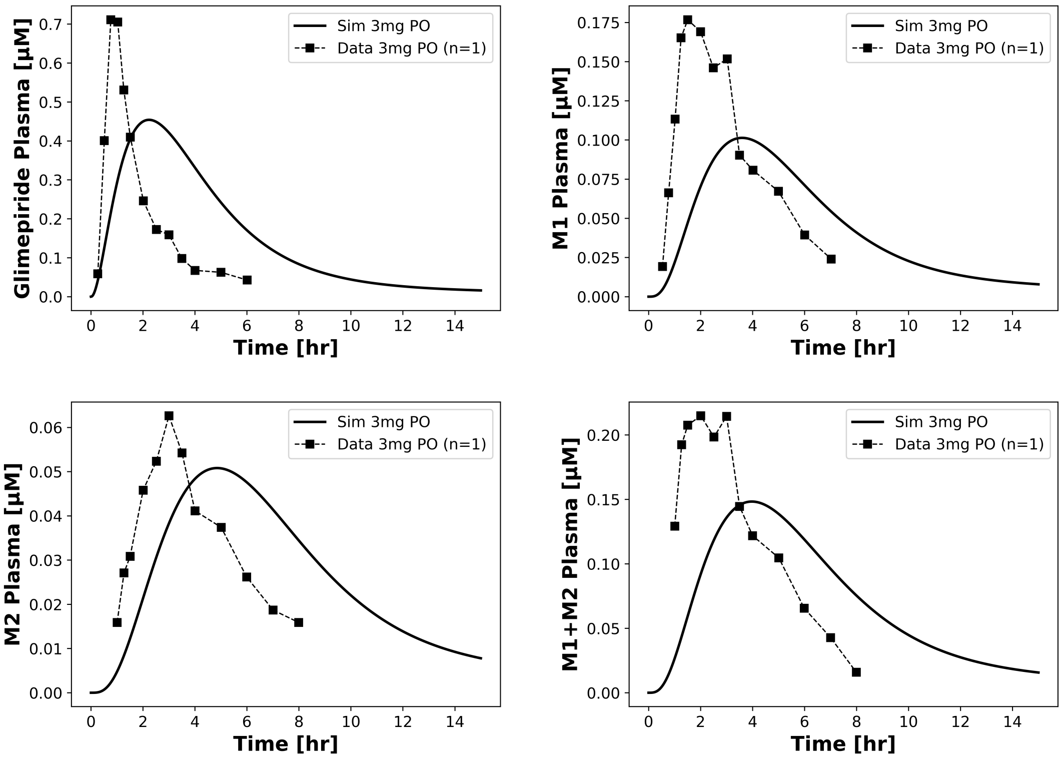

Figure S21: **Simulation Lehr1990 Lehr and Damm (1990).** Simulated versus observed plasma concentrations of glimepiride, M1, M2, and their sum (M1 + M2) after a 3 mg oral dose in a healthy volunteer. Data points represent the results from a single individual (n=1).

S6.11 Liu2010 (Fig. S22)

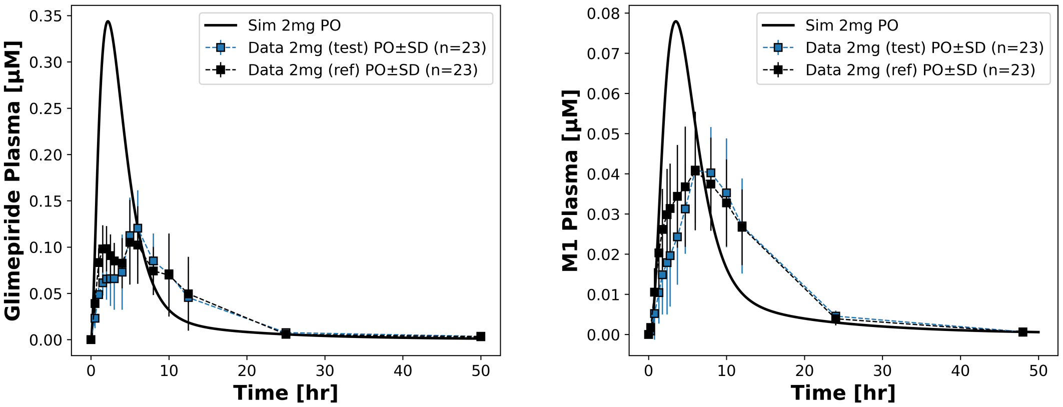

Figure S22: **Simulation Liu2010 Liu et al. (2010).** Simulated versus observed plasma concentrations of glimepiride and its metabolite M1 after a 2 mg oral dose in healthy Chinese volunteers (test and reference formulations). Error bars represent ±SD (n=23).

S6.12 Malerczyk1994 (Fig. S23)

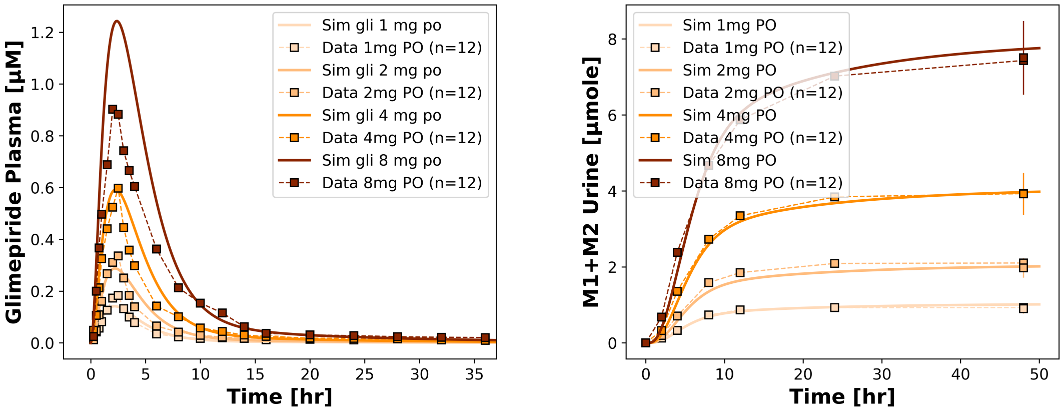

Figure S23: **Simulation Malerczyk1994 Malerczyk et al. (1994).** Simulated versus observed plasma concentrations of glimepiride and cumulative urinary excretion of its metabolites (M1 + M2) oral doses (1–8 mg) in healthy volunteers. Error bars represent ±SD (n=12).

S6.13 Matsuki2007 (Fig. S24)

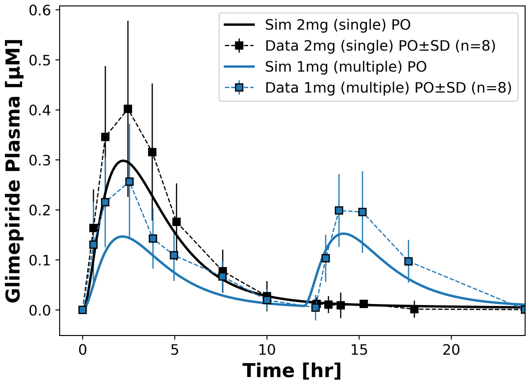

Figure S24: **Simulation Matsuki2007 Matsuki et al. (2007).** Simulated versus observed glimepiride plasma concentrations after a 2 mg single oral dose or two 1 mg doses in Japanese type 2 diabetic volunteers. Error bars represent ±SD (n=8).

S6.14 Niemi2002 (Fig. S25)

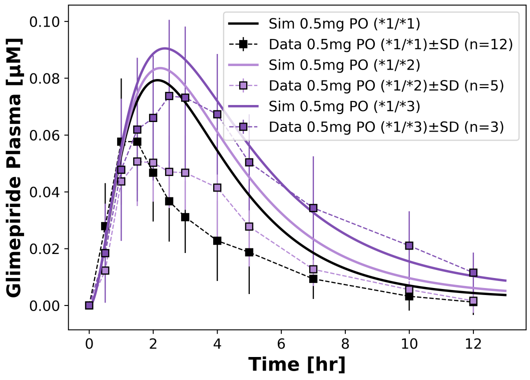

Figure S25: **Simulation Niemi2002 Niemi et al. (2002).** Simulated versus observed glimepiride plasma concentrations after a 0.5 mg single oral dose in Finnish volunteers with different CYP2C9 genotypes. Error bars represent ±SD.

S6.15 Ratheiser1993 (Fig. S26)

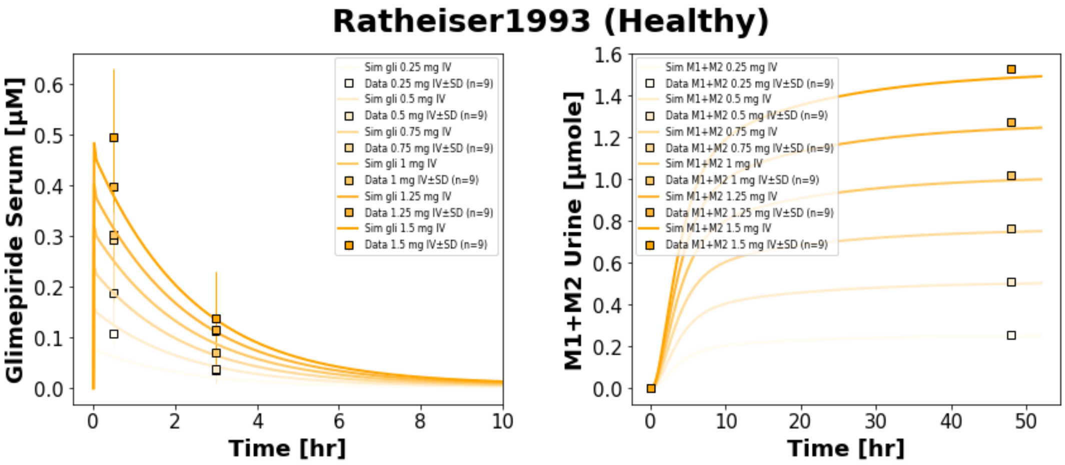

Figure S26: **Simulation Ratheiser1993 Ratheiser et al. (1993).** Simulated versus observed glimepiride plasma concentrations and cumulative urinary metabolite excretion after various single IV doses in healthy volunteers. Error bars represent ±SD.

S6.16 Rosenkranz1996a (Fig. S27 – S28)

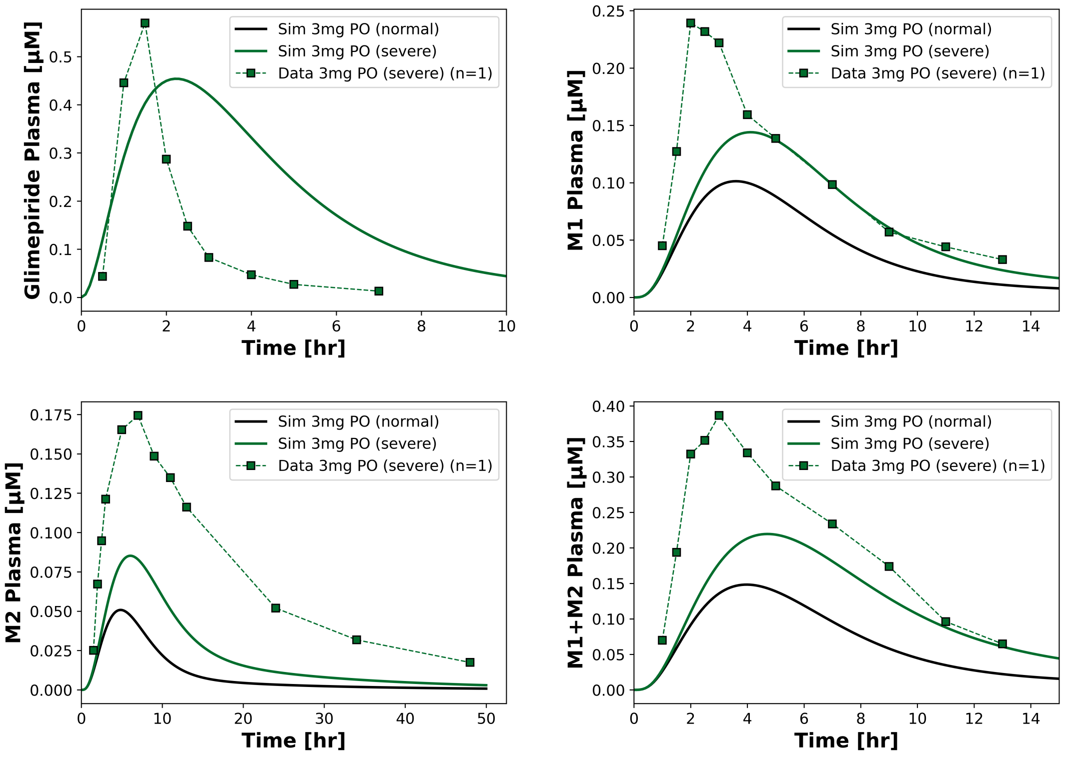

Figure S27: **Simulation Rosenkranz1996a Rosenkranz et al. (1996).** Simulated versus observed glimepiride, M1, M2 and M1 + M2 plasma concentrations and cumulative urinary metabolite excretion after a 3 mg single oral dose in a volunteer with severe renal impairment. Error bars represent ±SD.

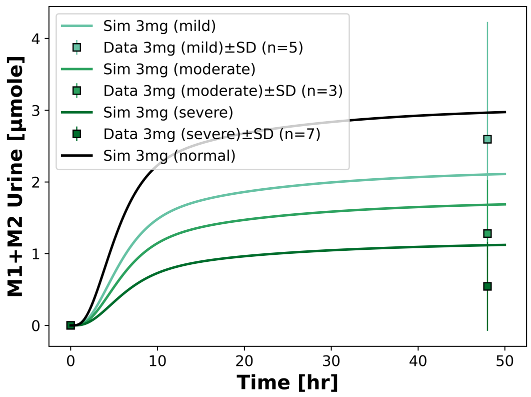

Figure S28: **Simulation Rosenkranz1996a Rosenkranz et al. (1996).** Simulated versus observed cumulative urinary metabolite excretion after a 3 mg single oral dose in volunteers with different degrees of renal impairment. Error bars represent ±SD.

S6.17 Shukla2004 (Fig. S29 – S30)

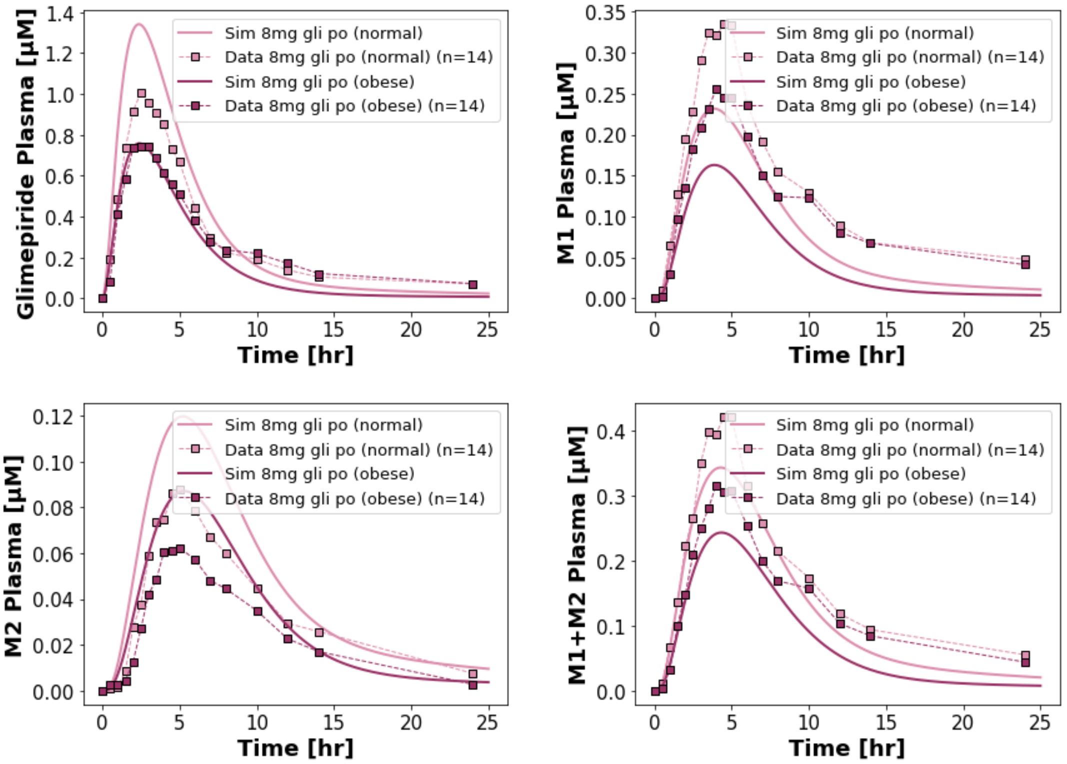

Figure S29: **Simulation Shukla2004 Shukla et al. (2004).** Simulated versus observed glimepiride, M1, M2 and M1 + M2 plasma concentrations after an 8 mg single oral dose in normal weight and morbidly obese volunteers. Error bars represent ±SD.

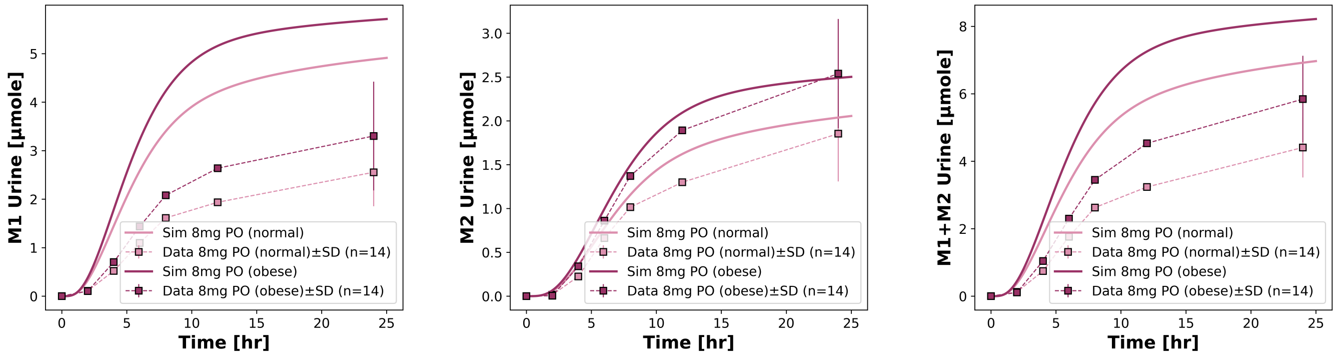

Figure S30: **Simulation Shukla2004 Shukla et al. (2004).** Simulated versus observed M1, M2 and M1 + M2 urinary excretion after an 8 mg single oral dose in normal weight and morbidly obese volunteers. Error bars represent ±SD.

S6.18 Suzuki2006 (Fig. S31)

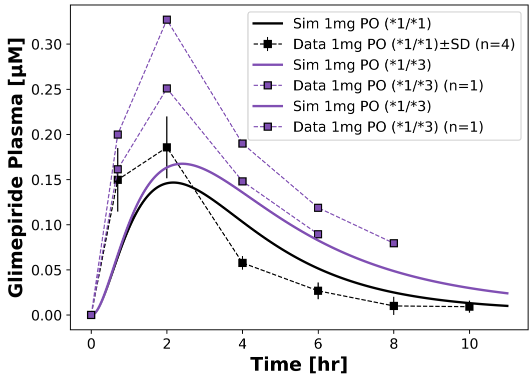

Figure S31: **Simulation Suzuki2006 Suzuki et al. (2006).** Simulated versus observed glimepiride plasma concentrations after a 1 mg single oral dose in Japanese volunteers with different CYP2C9 genotypes. Error bars represent ±SD.

S6.19 Wang2005 (Fig. S32)

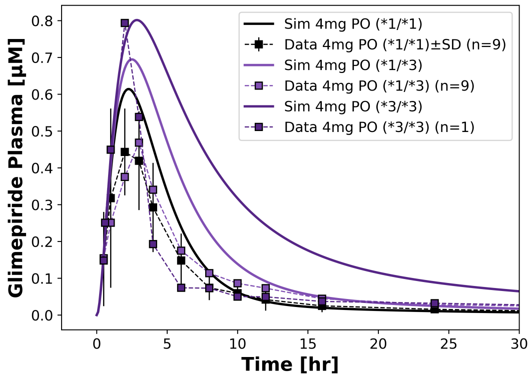

Figure S32: **Simulation Wang2005 Wang et al. (2005).** Simulated versus observed glimepiride plasma concentrations after a 4 mg single oral dose in Chinese volunteers with different CYP2C9 genotypes. Error bars represent ±SD.

S6.20 Yoo2011 (Fig. S33)

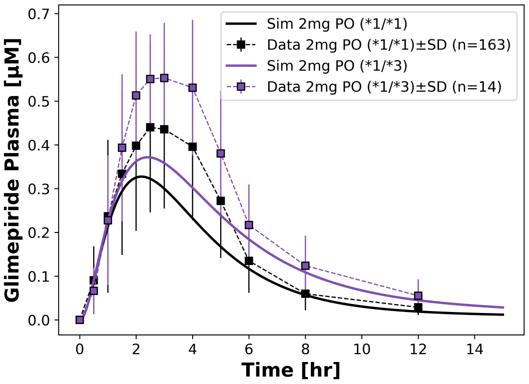

Figure S33: **Simulation Yoo2011 Yoo et al. (2011).** Simulated versus observed glimepiride plasma concentrations after a 2 mg single oral dose in Korean volunteers with different CYP2C9 genotypes. Error bars represent ±SD.

S7 CYP2C9 ANALYSIS

S7.1 Activity of CYP2C9 Alleles (Tab. S2)

Table S2. Relative enzymatic activity of CYP2C9 allelic variants derived from literature. Activity values are normalized to wild-type (∗1) activity.

Allele	Activity	References	Allele	Activity	References
∗1	1.00	Reference allele	∗38	0.64	Dai et al. (2014)
∗2	0.68	Dai et al. (2014), Yang et al. (2018)	∗39	0.10	Dai et al. (2014)
∗3	0.23	Dai et al. (2014), Yang et al. (2018), Maekawa et al. (2009), Suzuki et al. (2006)	∗40	1.03	Dai et al. (2014)
∗8	0.09	Dai et al. (2014)	∗41	0.75	Dai et al. (2014)
∗11	0.61	Dai et al. (2014)	∗42	0.03	Dai et al. (2014)
∗13	0.02	Dai et al. (2014)	∗43	0.07	Dai et al. (2014)
∗14	0.06	Dai et al. (2014)	∗44	0.15	Dai et al. (2014)
∗16	0.04	Dai et al. (2014)	∗45	0.08	Dai et al. (2014)
∗19	0.01	Dai et al. (2014)	∗46	0.23	Dai et al. (2014)
∗23	0.07	Dai et al. (2014)	∗47	1.15	Dai et al. (2014)
∗26	0.10	Dai et al. (2014)	∗48	0.67	Dai et al. (2014)
∗27	0.15	Dai et al. (2014)	∗49	0.51	Dai et al. (2014)
∗28	0.44	Dai et al. (2014)	∗50	0.29	Dai et al. (2014)
∗29	0.36	Dai et al. (2014)	∗51	0.91	Dai et al. (2014)
∗30	0.25	Dai et al. (2014)	∗52	0.03	Dai et al. (2014)
∗31	0.14	Dai et al. (2014)	∗53	0.87	Dai et al. (2014)
∗33	0.04	Dai et al. (2014)	∗54	0.95	Dai et al. (2014)
∗34	0.41	Dai et al. (2014)	∗55	0.11	Dai et al. (2014)
∗36	1.46	Dai et al. (2014)	∗56	0.81	Dai et al. (2014)
∗37	0.75	Dai et al. (2014)			

S7.2 CYP2C9 Intrinsic Clearance (Tab. S3)

Table S3. Statistical analysis of CYP2C9 intrinsic clearance for diclofenac in human liver microsomes. Data from Yang et al. Yang et al. (2012).

Statistic	Value	Unit
Mean	251.47	L/hr
Median	222.00	L/hr
SD	173.55	L/hr
Minimum	15.00	L/hr
Maximum	790.00	L/hr
First quartile (Q1)	103.75	L/hr
Third quartile (Q3)	331.25	L/hr
Sample size: 60		

S7.3 CYP2C9 Lognormal Distribution (Tab. S4)

Table S4. Parameters of fitted lognormal distribution based on CYP2C9 intrinsic clearance data from Yang et al. Yang et al. (2012).

Parameter	Value
Shape (σ)	0.81
Scale (s)	191.31
Arithmetic Mean	264.99
SD	253.98
Coefficient of Variation	0.96
Mode	99.72
Kolmogorov-Smirnov test D	0.10
Kolmogorov-Smirnov test p-value	0.55

S7.4 Sampled CYP2C9 Allele Activities (Tab. S5)

Table S5. Enzymatic activity of CYP2C9 allelic variants and genotypes derived from probabilistic sampling. Allelic activities are scaled relative to wild-type (∗1). Genotype activities represent combined effects of paired alleles.

Allele	Mean	Median	SD	Mode
∗1	1.00	1.00	0.73	0.91
∗2	0.68	0.68	0.49	0.63
∗3	0.23	0.23	0.17	0.21
Genotype	Mean	Median	SD	Mode
∗1/∗1	1.00	0.83	0.69	0.58
∗1/∗2	0.84	0.70	0.56	0.49
∗1/∗3	0.62	0.48	0.50	0.32
∗3/∗3	0.23	0.19	0.16	0.13
Sample size: 100,000				

S7.5 Sampled Glimepiride PK Parameters by CYP2C9 Genotype (Tab. S6)

Table S6. Sampled glimepiride pharmacokinetic parameters by CYP2C9 genotype

**Genotype**	**Mean**	**Median**	**Min**	**Q1**	**Q3**	**Max**
**Glimepiride AUC [ng*hr/ml]**						
∗1/∗1	1925.66	1864.73	307.07	1340.56	2427.87	4405.66
∗1/∗2	2196.06	2145.33	530.77	1614.88	2689.94	4885.69
∗1/∗3	2649.11	2644.96	226.37	1985.12	3353.00	4966.32
∗3/∗3	3842.99	3933.03	1272.23	3395.80	4358.30	5198.78
**Glimepiride T_{max} [hr]**						
∗1/∗1	2.32	2.35	1.35	2.13	2.52	2.97
∗1/∗2	2.42	2.44	1.61	2.26	2.60	3.05
∗1/∗3	2.55	2.58	1.23	2.39	2.76	3.07
∗3/∗3	2.85	2.88	2.10	2.76	2.96	3.11
**Glimepiride C_{max} [µM]**						
∗1/∗1	0.630	0.645	0.245	0.567	0.705	0.834
∗1/∗2	0.665	0.677	0.356	0.611	0.727	0.856
∗1/∗3	0.707	0.724	0.194	0.659	0.775	0.860
∗3/∗3	0.800	0.809	0.554	0.778	0.831	0.870

S7.6 Sampled M1 PK Parameters by CYP2C9 Genotype (Tab. S7)

Table S7. Sampled M1 pharmacokinetic parameters by CYP2C9 genotype

Genotype	Mean	Median	Min	Q1	Q3	Max
**M1 AUC [ng*hr/mL]**						
∗1/∗1	435.58	445.58	147.59	387.16	496.32	587.29
∗1/∗2	407.62	417.01	82.22	358.47	470.17	567.74
∗1/∗3	357.63	363.47	71.01	281.58	433.45	594.21
∗3/∗3	217.92	209.45	38.33	153.90	276.41	502.69
**M1 T_{max} [hr]**						
∗1/∗1	3.74	3.78	2.43	3.48	4.03	4.67
∗1/∗2	3.88	3.91	2.76	3.65	4.13	4.79
∗1/∗3	4.07	4.12	2.28	3.84	4.36	4.81
∗3/∗3	4.49	4.53	3.43	4.37	4.66	4.88
**M1 C_{max} [µM]**						
∗1/∗1	0.121	0.114	0.022	0.085	0.153	0.296
∗1/∗2	0.105	0.099	0.012	0.074	0.131	0.252
∗1/∗3	0.084	0.075	0.010	0.050	0.107	0.313
∗3/∗3	0.038	0.034	0.005	0.023	0.049	0.159

S7.7 Sampled M2 PK Parameters by CYP2C9 Genotype (Tab. S8)

Table S8. Sampled M2 pharmacokinetic parameters by CYP2C9 genotype

Genotype	Mean	Median	Min	Q1	Q3	Max
**M2 AUC [ng*hr/mL]**						
∗1/∗1	279.67	286.00	92.17	247.04	320.15	382.02
∗1/∗2	261.08	266.89	51.11	228.08	302.52	368.66
∗1/∗3	228.18	231.38	44.10	177.84	277.87	386.75
∗3/∗3	137.32	131.45	23.76	96.17	174.50	324.46
**M2 T_{max} [hr]**						
∗1/∗1	5.08	5.12	3.34	4.69	5.48	6.46
∗1/∗2	5.28	5.31	3.74	4.92	5.64	6.66
∗1/∗3	5.55	5.62	3.19	5.20	5.98	6.70
∗3/∗3	6.20	6.25	4.63	6.00	6.44	6.80
**M2 C_{max} [µM]**						
∗1/∗1	0.061	0.059	0.012	0.044	0.076	0.129
∗1/∗2	0.053	0.051	0.006	0.039	0.066	0.115
∗1/∗3	0.043	0.040	0.005	0.027	0.055	0.134
∗3/∗3	0.021	0.018	0.003	0.013	0.026	0.079

S8 BIOGEOGRAPHICAL GROUPS ANALYSIS

S8.1 AUC by Biogeographical Groups (Tab. S9)

Table S9. Mean (SD) for AUC [ng*hr/ml] of glimepiride and metabolites by biogeographical group

Ethnicity	Glimepiride	M1	M2
African American/Afro-Caribbean	2032.83 (858.49)	423.61 (90.79)	271.82 (60.12)
American	2060.73 (844.07)	420.92 (88.63)	270.00 (58.79)
Central/South Asian	2164.15 (842.47)	410.23 (89.37)	262.89 (59.18)
East Asian	2120.46 (863.78)	414.52 (91.77)	265.77 (60.75)
European	2150.44 (825.32)	411.85 (87.34)	263.94 (57.87)
Latino	2116.43 (846.06)	415.15 (89.48)	266.17 (59.27)
Near Eastern	2163.27 (876.49)	409.93 (93.08)	262.73 (61.62)
Oceanian	1989.97 (773.72)	428.92 (81.22)	275.26 (53.89)
Sub-Saharan African	2174.18 (876.54)	408.80 (94.93)	262.01 (62.59)

S8.2 T_{max} by Biogeographical Groups (Tab. S10)

Table S10. Mean (SD) for T_{max} [hr] of glimepiride and metabolites by biogeographical groups

Biogeographical Group	Glimepiride	M1	M2
African American/Afro-Caribbean	2.35 (0.30)	3.79 (0.42)	5.15 (0.60)
American	2.36 (0.30)	3.80 (0.42)	5.17 (0.60)
Central/South Asian	2.40 (0.29)	3.85 (0.40)	5.24 (0.58)
East Asian	2.38 (0.29)	3.83 (0.41)	5.21 (0.59)
European	2.40 (0.28)	3.85 (0.39)	5.24 (0.57)
Latino	2.38 (0.29)	3.83 (0.41)	5.21 (0.59)
Near Eastern	2.40 (0.30)	3.85 (0.42)	5.23 (0.61)
Oceanian	2.35 (0.27)	3.78 (0.38)	5.13 (0.54)
Sub-Saharan African	2.40 (0.29)	3.86 (0.40)	5.25 (0.58)

S8.3 C_{max} by Biogeographical Groups (Tab. S11)

Table S11. Mean (SD) for C_{max} [µM] of glimepiride and metabolites by biogeographical groups

Biogeographical Group	Glimepiride	M1	M2
African American/Afro-Caribbean	0.64 (0.11)	0.1164 (0.0518)	0.0584 (0.0234)
American	0.64 (0.11)	0.1146 (0.0514)	0.0575 (0.0232)
Central/South Asian	0.66 (0.10)	0.1081 (0.0490)	0.0546 (0.0224)
East Asian	0.65 (0.10)	0.1111 (0.0501)	0.0560 (0.0229)
European	0.66 (0.10)	0.1085 (0.0481)	0.0548 (0.0220)
Latino	0.65 (0.10)	0.1112 (0.0505)	0.0560 (0.0229)
Near Eastern	0.66 (0.11)	0.1092 (0.0517)	0.0550 (0.0234)
Oceanian	0.64 (0.09)	0.1168 (0.0464)	0.0588 (0.0211)
Sub-Saharan African	0.66 (0.10)	0.1077 (0.0484)	0.0544 (0.0222)

S8.4 Most Significant Differences by Biogeographical Groups (Tab. S12)

Table S12. Most significant differences in PK parameters

Compound	Parameter	Group 1	Group 2	KS Stat	Adj. p-value	Sig.	% Diff
Glimepiride	AUC	Near Eastern	Oceanian	0.12	5.37×10^{-5}	***	-9.7%
Glimepiride	AUC	African American	Near Eastern	0.10	1.45×10^{-3}	**	10.0%
Glimepiride	AUC	African American	European	0.09	3.53×10^{-3}	**	9.1%
M1	Cmax	Near Eastern	Oceanian	0.12	5.37×10^{-5}	***	11.6%
M1	Cmax	Central/South Asian	Oceanian	0.12	5.37×10^{-5}	***	10.6%
M1	Cmax	African American	Near Eastern	0.10	1.45×10^{-3}	**	-9.7%
M1	Cmax	African American	European	0.09	3.53×10^{-3}	**	-8.9%
M2	Cmax	Near Eastern	Oceanian	0.12	5.37×10^{-5}	***	10.6%
M2	Cmax	Central/South Asian	Oceanian	0.12	5.37×10^{-5}	***	9.7%
M2	Cmax	African American	Near Eastern	0.10	1.45×10^{-3}	**	-9.0%
M2	Cmax	African American	European	0.09	3.53×10^{-3}	**	-8.2%
M2	AUC	Near Eastern	Oceanian	0.12	5.37×10^{-5}	***	5.2%
M2	Tmax	Near Eastern	Oceanian	0.12	4.51×10^{-5}	***	-2.7%
M2	Tmax	African American	Near Eastern	0.10	8.72×10^{-4}	***	2.6%

Notes: Significance: *** $p < 0.001$, ** $p < 0.01$. KS Stat = Kolmogorov-Smirnov statistic (maximum difference between distribution functions). The percentage difference shows the relative difference between median values. P-values were adjusted using the Benjamini-Hochberg false discovery rate (FDR) procedure to address multiple comparisons across ethnic groups and pharmacokinetic parameters. African American refers to African American/Afro-Caribbean ethnicity.

S8.5 Sampled CYP2C9 Genotypes Freqiencies by Biogeographical Groups (Tab. S13)

Table S13: Sampled CYP2C9 genotypes and their resulting frequencies in biogeographical populations. Only genotypes with known enzymatic activity values from the literature are included.

Genotype	AA	AM	CA	EA	EU	LA	NE	OC	SA	Activity
*1/*1	0.7587	0.8315	0.5962	0.8379	0.6285	0.7434	0.6113	0.9122	0.5264	1.00
*1/*2	0.0391	0.0609	0.1757	0.0039	0.2018	0.1315	0.2029	0.056	0.019	0.84
*1/*3	0.0235	0.0548	0.1696	0.0689	0.1198	0.0692	0.129	0.0298	0.0162	0.62
*1/*8	0.1028	0.0372	0.0015	0.0068	0.0029	0.0127	0.001	0	0.1101	0.55
*1/*11	0.0241	0.0051	0.0016	0.0005	0.0026	0.005	0	0	0.0373	0.81
*2/*3	0.0006	0.002	0.025	0.0002	0.0192	0.0061	0.0214	0.0009	0.0003	0.46
*2/*2	0.0005	0.0011	0.0129	4.00e-06	0.0162	0.0058	0.0168	0.0009	0.0002	0.68
*3/*3	0.0002	0.0009	0.0121	0.0014	0.0057	0.0016	0.0068	0.0002	0.0001	0.23
*1/*42	0	0	0	0.0183	0	0.0002	0.0009	0	0	0.52
*1/*55	0	0	0	0.019	0	0	0	0	0	0.56
*8/*8	0.0035	0.0004	0	1.30e-05	3.00e-06	5.40e-05	0	0	0.0058	0.09
*2/*8	0.0026	0.0014	0.0002	1.50e-05	0.0005	0.0011	0.0002	0	0.002	0.39
*1/*13	0	0	0	0.0061	0	0	0	0	0	0.51
*3/*8	0.0016	0.0012	0.0002	0.0003	0.0003	0.0006	0.0001	0	0.0017	0.16
*8/*11	0.0016	0.0001	2.00e-06	2.00e-06	5.00e-06	4.20e-05	0	0	0.0039	0.35
*1/*29	0	0	0	0.004	0	0	0	0	0	0.68
*1/*26	0	0	0	0.0037	0	0	0	0	0	0.55
*1/*28	0	0	0	0.0037	0	0	0	0	0	0.72
*1/*16	0	0	0	0.0034	0	0	0	0	0	0.52
*1/*27	0	0	0	0.003	0	0	0	0	0	0.58
*1/*31	0	0	0	0.0029	0	0	0	0	0	0.57
*1/*30	0	0	0	0.0029	0	0	0	0	0	0.63
*1/*33	0	0	0	0.0012	0	0	0.0016	0	0	0.52
*2/*11	0.0006	0.0002	0.0002	1.00e-06	0.0004	0.0004	0	0	0.0007	0.65
*1/*14	0	0	0	0.0006	0	0	0.0016	0	0	0.53
*3/*11	0.0004	0.0002	0.0002	2.20e-05	0.0002	0.0002	0	0	0.0006	0.42
*1/*45	0	0	0.0003	0	0.0003	0.0009	0	0	0	0.54
*1/*19	0	0	0	0.0009	0	0	0	0	0	0.51
*11/*11	0.0002	7.00e-06	1.00e-06	0	2.00e-06	8.00e-06	0	0	0.0007	0.61
*3/*42	0	0	0	0.0008	0	8.00e-06	8.90e-05	0	0	0.13
*3/*55	0	0	0	0.0008	0	0	0	0	0	0.17
*1/*44	0	0	0	0	0	0.0005	0	0	0	0.58
*1/*34	0	0	0	0.0004	0	0	0	0	0	0.71
*1/*23	0	0	0	0.0004	0	0	0	0	0	0.54
*2/*33	0	0	0	2.00e-06	0	0	0.0003	0	0	0.36
*2/*14	0	0	0	1.00e-06	0	0	0.0003	0	0	0.37
*3/*13	0	0	0	0.0002	0	0	0	0	0	0.13
*3/*33	0	0	0	4.90e-05	0	0	0.0002	0	0	0.14
*42/*55	0	0	0	0.0002	0	0	0	0	0	0.07
*2/*42	0	0	0	4.20e-05	0	1.50e-05	0.0001	0	0	0.36
*3/*14	0	0	0	2.50e-05	0	0	0.0002	0	0	0.15
*2/*45	0	0	4.50e-05	0	5.00e-05	7.60e-05	0	0	0	0.38

Continued on next page

Table S13 – continued from previous page

Genotype	AA	AM	CA	EA	EU	LA	NE	OC	SA	Activity
*3/*29	0	0	0	0.0002	0	0	0	0	0	0.30
*1/*43	0	0	0.0002	0	0	0	0	0	0	0.54
*3/*28	0	0	0	0.0001	0	0	0	0	0	0.34
*3/*26	0	0	0	0.0001	0	0	0	0	0	0.17
*3/*16	0	0	0	0.0001	0	0	0	0	0	0.14
*3/*27	0	0	0	0.0001	0	0	0	0	0	0.19
*3/*31	0	0	0	0.0001	0	0	0	0	0	0.19
*3/*30	0	0	0	0.0001	0	0	0	0	0	0.24
*3/*45	0	0	4.30e-05	0	3.00e-05	4.00e-05	0	0	0	0.16
*55/*55	0	0	0	0.0001	0	0	0	0	0	0.11
*42/*42	0	0	0	0.0001	0	0	0	0	0	0.03
*8/*55	0	0	0	7.70e-05	0	0	0	0	0	0.10
*8/*42	0	0	0	7.40e-05	0	1.00e-06	0	0	0	0.06
*13/*55	0	0	0	6.80e-05	0	0	0	0	0	0.07
*13/*42	0	0	0	6.60e-05	0	0	0	0	0	0.03
*2/*44	0	0	0	0	0	4.50e-05	0	0	0	0.42
*29/*55	0	0	0	4.50e-05	0	0	0	0	0	0.24
*2/*55	0	0	0	4.40e-05	0	0	0	0	0	0.40
*29/*42	0	0	0	4.40e-05	0	0	0	0	0	0.20
*26/*55	0	0	0	4.10e-05	0	0	0	0	0	0.11
*28/*55	0	0	0	4.10e-05	0	0	0	0	0	0.28
*26/*42	0	0	0	4.00e-05	0	0	0	0	0	0.07
*28/*42	0	0	0	4.00e-05	0	0	0	0	0	0.24
*16/*55	0	0	0	3.90e-05	0	0	0	0	0	0.08
*16/*42	0	0	0	3.70e-05	0	0	0	0	0	0.04
*3/*19	0	0	0	3.50e-05	0	0	0	0	0	0.12
*27/*55	0	0	0	3.30e-05	0	0	0	0	0	0.13
*31/*55	0	0	0	3.30e-05	0	0	0	0	0	0.13
*27/*42	0	0	0	3.20e-05	0	0	0	0	0	0.09
*31/*42	0	0	0	3.20e-05	0	0	0	0	0	0.09
*30/*55	0	0	0	3.20e-05	0	0	0	0	0	0.18
*30/*42	0	0	0	3.10e-05	0	0	0	0	0	0.14
*8/*13	0	0	0	2.40e-05	0	0	0	0	0	0.06
*3/*44	0	0	0	0	0	2.40e-05	0	0	0	0.19
*2/*43	0	0	2.20e-05	0	0	0	0	0	0	0.38
*3/*43	0	0	2.10e-05	0	0	0	0	0	0	0.15
*8/*29	0	0	0	1.60e-05	0	0	0	0	0	0.23
*3/*23	0	0	0	1.50e-05	0	0	0	0	0	0.15
*3/*34	0	0	0	1.50e-05	0	0	0	0	0	0.32
*8/*26	0	0	0	1.40e-05	0	0	0	0	0	0.10
*8/*28	0	0	0	1.40e-05	0	0	0	0	0	0.27
*2/*13	0	0	0	1.40e-05	0	0	0	0	0	0.35
*13/*29	0	0	0	1.40e-05	0	0	0	0	0	0.19
*33/*42	0	0	0	1.30e-05	0	0	1.00e-06	0	0	0.04
*8/*16	0	0	0	1.40e-05	0	0	0	0	0	0.07
*33/*55	0	0	0	1.30e-05	0	0	0	0	0	0.08
*13/*28	0	0	0	1.30e-05	0	0	0	0	0	0.23

Continued on next page

Table S13 – continued from previous page

Genotype	AA	AM	CA	EA	EU	LA	NE	OC	SA	Activity
*13/*26	0	0	0	1.30e-05	0	0	0	0	0	0.06
*13/*16	0	0	0	1.20e-05	0	0	0	0	0	0.03
*8/*27	0	0	0	1.20e-05	0	0	0	0	0	0.12
*13/*13	0	0	0	1.10e-05	0	0	0	0	0	0.02
*8/*31	0	0	0	1.10e-05	0	0	0	0	0	0.12
*8/*30	0	0	0	1.10e-05	0	0	0	0	0	0.17
*13/*27	0	0	0	1.00e-05	0	0	0	0	0	0.09
*13/*30	0	0	0	1.00e-05	0	0	0	0	0	0.14
*13/*31	0	0	0	1.00e-05	0	0	0	0	0	0.08
*19/*42	0	0	0	9.00e-06	0	0	0	0	0	0.02
*19/*55	0	0	0	9.00e-06	0	0	0	0	0	0.06
*2/*29	0	0	0	9.00e-06	0	0	0	0	0	0.52
*26/*28	0	0	0	8.00e-06	0	0	0	0	0	0.27
*26/*29	0	0	0	8.00e-06	0	0	0	0	0	0.23
*28/*29	0	0	0	8.00e-06	0	0	0	0	0	0.40
*2/*26	0	0	0	8.00e-06	0	0	0	0	0	0.39
*16/*29	0	0	0	8.00e-06	0	0	0	0	0	0.20
*2/*28	0	0	0	8.00e-06	0	0	0	0	0	0.56
*27/*29	0	0	0	7.00e-06	0	0	0	0	0	0.26
*14/*42	0	0	0	6.00e-06	0	0	1.00e-06	0	0	0.05
*29/*31	0	0	0	7.00e-06	0	0	0	0	0	0.25
*16/*28	0	0	0	7.00e-06	0	0	0	0	0	0.24
*16/*26	0	0	0	7.00e-06	0	0	0	0	0	0.07
*2/*16	0	0	0	7.00e-06	0	0	0	0	0	0.36
*8/*45	0	0	0	0	0	7.00e-06	0	0	0	0.09
*16/*31	0	0	0	6.00e-06	0	0	0	0	0	0.09
*2/*30	0	0	0	6.00e-06	0	0	0	0	0	0.47
*2/*31	0	0	0	6.00e-06	0	0	0	0	0	0.41
*2/*27	0	0	0	6.00e-06	0	0	0	0	0	0.42
*29/*30	0	0	0	6.00e-06	0	0	0	0	0	0.31
*14/*55	0	0	0	6.00e-06	0	0	0	0	0	0.09
*28/*30	0	0	0	6.00e-06	0	0	0	0	0	0.35
*28/*31	0	0	0	6.00e-06	0	0	0	0	0	0.29
*26/*27	0	0	0	6.00e-06	0	0	0	0	0	0.13
*26/*30	0	0	0	6.00e-06	0	0	0	0	0	0.18
*26/*31	0	0	0	6.00e-06	0	0	0	0	0	0.12
*27/*28	0	0	0	6.00e-06	0	0	0	0	0	0.30
*11/*55	0	0	0	6.00e-06	0	0	0	0	0	0.36
*16/*27	0	0	0	6.00e-06	0	0	0	0	0	0.10
*27/*31	0	0	0	5.00e-06	0	0	0	0	0	0.15
*27/*30	0	0	0	5.00e-06	0	0	0	0	0	0.20
*16/*30	0	0	0	5.00e-06	0	0	0	0	0	0.15
*11/*42	0	0	0	5.00e-06	0	0	0	0	0	0.32
*30/*31	0	0	0	5.00e-06	0	0	0	0	0	0.20
*8/*33	0	0	0	4.00e-06	0	0	1.00e-06	0	0	0.07
*34/*42	0	0	0	4.00e-06	0	0	0	0	0	0.22
*34/*55	0	0	0	4.00e-06	0	0	0	0	0	0.26

Continued on next page

Table S13 – continued from previous page

Genotype	AA	AM	CA	EA	EU	LA	NE	OC	SA	Activity
*13/*33	0	0	0	4.00e-06	0	0	0	0	0	0.03
*8/*44	0	0	0	0	0	4.00e-06	0	0	0	0.12
*23/*55	0	0	0	4.00e-06	0	0	0	0	0	0.09
*23/*42	0	0	0	4.00e-06	0	0	0	0	0	0.05
*28/*28	0	0	0	4.00e-06	0	0	0	0	0	0.44
*29/*29	0	0	0	4.00e-06	0	0	0	0	0	0.36
*26/*26	0	0	0	4.00e-06	0	0	0	0	0	0.10
*8/*14	0	0	0	2.00e-06	0	0	1.00e-06	0	0	0.08
*8/*19	0	0	0	3.00e-06	0	0	0	0	0	0.05
*16/*16	0	0	0	3.00e-06	0	0	0	0	0	0.04
*13/*19	0	0	0	3.00e-06	0	0	0	0	0	0.02
*13/*14	0	0	0	2.00e-06	0	0	0	0	0	0.04
*26/*33	0	0	0	2.00e-06	0	0	0	0	0	0.07
*16/*33	0	0	0	2.00e-06	0	0	0	0	0	0.04
*27/*27	0	0	0	2.00e-06	0	0	0	0	0	0.15
*11/*45	0	0	0	0	0	2.00e-06	0	0	0	0.35
*31/*33	0	0	0	2.00e-06	0	0	0	0	0	0.09
*31/*31	0	0	0	2.00e-06	0	0	0	0	0	0.14
*27/*33	0	0	0	2.00e-06	0	0	0	0	0	0.10
*30/*33	0	0	0	2.00e-06	0	0	0	0	0	0.15
*30/*30	0	0	0	2.00e-06	0	0	0	0	0	0.25
*29/*33	0	0	0	2.00e-06	0	0	0	0	0	0.20
*19/*29	0	0	0	2.00e-06	0	0	0	0	0	0.19
*14/*33	0	0	0	0	0	0	2.00e-06	0	0	0.05
*2/*19	0	0	0	2.00e-06	0	0	0	0	0	0.35
*28/*33	0	0	0	2.00e-06	0	0	0	0	0	0.24
*19/*28	0	0	0	1.00e-06	0	0	0	0	0	0.23
*19/*30	0	0	0	1.00e-06	0	0	0	0	0	0.13
*19/*27	0	0	0	1.00e-06	0	0	0	0	0	0.08
*19/*26	0	0	0	1.00e-06	0	0	0	0	0	0.06
*8/*34	0	0	0	1.00e-06	0	0	0	0	0	0.25
*11/*16	0	0	0	1.00e-06	0	0	0	0	0	0.33
*19/*31	0	0	0	1.00e-06	0	0	0	0	0	0.08
*8/*23	0	0	0	1.00e-06	0	0	0	0	0	0.08
*11/*13	0	0	0	1.00e-06	0	0	0	0	0	0.32
*13/*34	0	0	0	1.00e-06	0	0	0	0	0	0.22
*11/*26	0	0	0	1.00e-06	0	0	0	0	0	0.36
*11/*28	0	0	0	1.00e-06	0	0	0	0	0	0.53
*11/*29	0	0	0	1.00e-06	0	0	0	0	0	0.49
*11/*44	0	0	0	0	0	1.00e-06	0	0	0	0.38
*14/*14	0	0	0	0	0	0	1.00e-06	0	0	0.06
*16/*19	0	0	0	1.00e-06	0	0	0	0	0	0.03
*33/*33	0	0	0	0	0	0	1.00e-06	0	0	0.04
*13/*23	0	0	0	1.00e-06	0	0	0	0	0	0.05
*14/*31	0	0	0	1.00e-06	0	0	0	0	0	0.10
*14/*30	0	0	0	1.00e-06	0	0	0	0	0	0.16
*14/*29	0	0	0	1.00e-06	0	0	0	0	0	0.21

Continued on next page

Table S13 – continued from previous page

Genotype	AA	AM	CA	EA	EU	LA	NE	OC	SA	Activity
*14/*27	0	0	0	1.00e-06	0	0	0	0	0	0.11
*14/*26	0	0	0	1.00e-06	0	0	0	0	0	0.08
*14/*16	0	0	0	1.00e-06	0	0	0	0	0	0.05
*14/*28	0	0	0	1.00e-06	0	0	0	0	0	0.25
Total	0.960	0.997	0.996	0.995	0.999	0.980	0.995	1	0.725	

AA: African American/Afro-Caribbean; AM: American; CA: Central/South Asian; EA: East Asian; EU: European; LA: Latino; NE: Near Eastern; OC: Oceanian; SA: Sub-Saharan African.

REFERENCES

[Dataset] Elias M, König M. Physiologically based pharmacokinetic (PBPK) model of glimepiride. Zenodo (2025). doi:10.5281/zenodo.15189579.

Ahmed TA, El-Say KM, Aljaeid BM, Fahmy UA, Abd-Allah FI. Transdermal glimepiride delivery system based on optimized ethosomal nano-vesicles: Preparation, characterization, in vitro, ex vivo and clinical evaluation. *International journal of pharmaceutics* **500** (2016) 245–254. doi:10.1016/j.ijpharm.2016.01.017.

Badian M, Korn A, Lehr KH, Malerczyk V, Waldhäusl W. Absolute bioavailability of glimepiride (Amaryl) after oral administration. *Drug metabolism and drug interactions* **11** (1994) 331–339. doi:10.1515/dmdi.1994.11.4.331.

Badian M, Korn A, Lehr KH, Malerczyk V, Waldhäusl W. Pharmacokinetics and pharmacodynamics of the hydroxymetabolite of glimepiride (Amaryl) after intravenous administration. *Drug metabolism and drug interactions* **13** (1996) 69–85. doi:10.1515/dmdi.1996.13.1.69.

Choi HY, Kim YH, Kim MJ, Lee SH, Bang K, Han S, et al. Evaluation of pharmacokinetic drug interactions between gemigliptin (dipeptidylpeptidase-4 inhibitor) and glimepiride (sulfonylurea) in healthy volunteers. *Drugs in R&D* **14** (2014) 165–176. doi:10.1007/s40268-014-0054-8.

US Food and Drug Administration (FDA). Glimepiride Drug Label – FDA Approved Information. Tech. rep., U.S. Food and Drug Administration (FDA) (1995).

Helmy SA, El Bedaiwy HM, Mansour NO. Dose Linearity of Glimepiride in Healthy Human Egyptian Volunteers. *Clinical pharmacology in drug development* **2** (2013) 264–269. doi:10.1002/cpdd.20.

Kasichayanula S, Liu X, Shyu WC, Zhang W, Pfister M, Griffen SC, et al. Lack of pharmacokinetic interaction between dapagliflozin, a novel sodium-glucose transporter 2 inhibitor, and metformin, pioglitazone, glimepiride or sitagliptin in healthy subjects. *Diabetes, obesity & metabolism* **13** (2011) 47–54. doi:10.1111/j.1463-1326.2010.01314.x.

Kim CO, Oh ES, Kim H, Park MS. Pharmacokinetic interactions between glimepiride and rosuvastatin in healthy Korean subjects: Does the SLCO1B1 or CYP2C9 genetic polymorphism affect these drug interactions? *Drug design, development and therapy* **11** (2017) 503–512. doi:10.2147/DDDT.S129586.

Lee HW, Lim Ms, Lee J, Jegal MY, Kim DW, Lee WK, et al. Frequency of CYP2C9 variant alleles, including CYP2C9*13 in a Korean population and effect on glimepiride pharmacokinetics. *Journal of clinical pharmacy and therapeutics* **37** (2012) 105–111. doi:10.1111/j.1365-2710.2010.01238.x.

Lehr KH, Damm P. Simultaneous determination of the sulphonylurea glimepiride and its metabolites in human serum and urine by high-performance liquid chromatography after pre-column derivatization. *Journal of chromatography* **526** (1990) 497–505. doi:10.1016/s0378-4347(00) 82531-1.

Liu Y, Zhang Mq, Zhu Jm, Jia Jy, Liu Ym, Liu Gy, et al. Bioequivalence and pharmacokinetic evaluation of two formulations of glimepiride 2 mg: A single-dose, randomized-sequence, open-label, two-way crossover study in healthy Chinese male volunteers. *Clinical therapeutics* **32** (2010) 986–995. doi:10.1016/j.clinthera.2010.04.016.

Malerczyk V, Badian M, Korn A, Lehr KH, Waldhäusl W. Dose linearity assessment of glimepiride (Amaryl) tablets in healthy volunteers. *Drug metabolism and drug interactions* **11** (1994) 341–357. doi:10.1515/dmdi.1994.11.4.341.

Matsuki M, Matsuda M, Kohara K, Shimoda M, Kanda Y, Tawaramoto K, et al. Pharmacokinetics and pharmacodynamics of glimepiride in type 2 diabetic patients: Compared effects of once- versus twice-daily dosing. *Endocrine journal* **54** (2007) 571–576. doi:10.1507/endocrj.k06-052.

Niemi M, Cascorbi I, Timm R, Kroemer HK, Neuvonen PJ, Kivistö KT. Glyburide and glimepiride pharmacokinetics in subjects with different CYP2C9 genotypes. *Clinical pharmacology and therapeutics* **72** (2002) 326–332. doi:10.1067/mcp.2002.127495.

Ratheiser K, Korn A, Waldhäusl W, Komjati M, Vierhapper H, Badian M, et al. Dose relationship of stimulated insulin production following intravenous application of glimepiride in healthy man. *Arzneimittel-Forschung* **43** (1993) 856–858.

Rosenkranz B, Profozic V, Metelko Z, Mrzljak V, Lange C, Malerczyk V. Pharmacokinetics and safety of glimepiride at clinically effective doses in diabetic patients with renal impairment. *Diabetologia* **39** (1996) 1617–1624. doi:10.1007/s001250050624.

Shukla UA, Chi EM, Lehr KH. Glimepiride pharmacokinetics in obese versus non-obese diabetic patients. *The Annals of pharmacotherapy* **38** (2004) 30–35. doi:10.1345/aph.1C397.

Suzuki K, Yanagawa T, Shibasaki T, Kaniwa N, Hasegawa R, Tohkin M. Effect of CYP2C9 genetic polymorphisms on the efficacy and pharmacokinetics of glimepiride in subjects with type 2 diabetes. *Diabetes research and clinical practice* **72** (2006) 148–154. doi:10.1016/j.diabres.2005.09.019.

Wang R, Chen K, Wen Sy, Li J, Wang Sq. Pharmacokinetics of glimepiride and cytochrome P450 2C9 genetic polymorphisms. *Clinical pharmacology and therapeutics* **78** (2005) 90–92. doi:10.1016/j.clpt.2005.03.008.

Yoo HD, Kim MS, Cho HY, Lee YB. Population pharmacokinetic analysis of glimepiride with CYP2C9 genetic polymorphism in healthy Korean subjects. *European journal of clinical pharmacology* **67** (2011) 889–898. doi:10.1007/s00228-011-1035-2.

Dai DP, Wang SH, Geng PW, Hu GX, Cai JP. *In Vitro* Assessment of 36 CYP 2 C 9 Allelic Isoforms Found in the C hinese Population on the Metabolism of Glimepiride. *Basic & Clinical Pharmacology & Toxicology* **114** (2014) 305–310. doi:10.1111/bcpt.12159.

Yang F, Xiong X, Liu Y, Zhang H, Huang S, Xiong Y, et al. CYP2C9 and OATP1B1 genetic polymorphisms affect the metabolism and transport of glimepiride and gliclazide. *Scientific reports* **8** (2018) 10994. doi:10.1038/s41598-018-29351-4.

Maekawa K, Harakawa N, Sugiyama E, Tohkin M, Kim SR, Kaniwa N, et al. Substrate-dependent functional alterations of seven CYP2C9 variants found in Japanese subjects. *Drug Metabolism and Disposition: The Biological Fate of Chemicals* **37** (2009) 1895–1903. doi:10.1124/dmd.109.027003.

Yang J, He MM, Niu W, Wrighton SA, Li L, Liu Y, et al. Metabolic capabilities of cytochrome P450 enzymes in Chinese liver microsomes compared with those in Caucasian liver microsomes. *British Journal of Clinical Pharmacology* **73** (2012) 268–284. doi:10.1111/j.1365-2125.2011.04076.x.
